# Inhibition of placental trophoblast fusion by guanylate-binding protein 5

**DOI:** 10.1126/sciadv.adt5388

**Published:** 2025-05-07

**Authors:** Veronika Krchlikova, Elisabeth Braun, Johanna Weiss, Krystof Stafl, Lukas Jech, Smitha Srinivasachar Badarinarayan, Rishikesh Lotke, Martin Travnicek, Charlotte Baur, Paul Stark, Isabell Haussmann, Yueshuang Lu, Moritz Petersen, Wen Cui, Wei Wang, Bianca M. Fäger, Hannah Reisinger, Kenzo Tokunaga, Oya Cingöz, Konstantin M. J. Sparrer, Madhuri S. Salker, Jiri Hejnar, Frank Kirchhoff, Katerina Trejbalova, Daniel Sauter

**Affiliations:** ^1^ Institute for Medical Virology, University Hospital Tübingen, Tübingen, Germany.; ^2^ Institute of Molecular Virology, Ulm University Medical Center, Ulm, Germany.; ^3^ Institute of Molecular Genetics of the Czech Academy of Sciences, Prague, Czechia.; ^4^ College of Pharmacy, Chongqing Medical University, Chongqing, China.; ^5^ Department of Pathology, National Institute of Infectious Diseases, Tokyo, Japan.; ^6^ Charité–Universitätsmedizin Berlin, Institute of Virology, Berlin, Germany.; ^7^ MRC–University of Glasgow, Centre for Virus Research (CVR), Glasgow, UK.; ^8^ German Center for Neurodegenerative Diseases (DZNE), Ulm, Germany.; ^9^ Research Institute for Women’s Health, University Hospital Tübingen, Tübingen, Germany.

## Abstract

Syncytin-1 and Syncytin-2 are envelope glycoproteins encoded by human endogenous retroviruses that have been exapted for the fusion of cytotrophoblast cells into syncytiotrophoblasts during placental development. Pregnancy complications like preeclampsia are associated with altered expression of interferon-stimulated genes, including guanylate-binding protein 5 (GBP5). Here, we show that misdirected antiviral activity of GBP5 impairs processing and activation of Syncytin-1. In contrast, the proteolytic activation of Syncytin-2 is not affected by GBP5, and its fusogenic activity is only modestly reduced. Mechanistic analyses revealed that Syncytin-1 is mainly cleaved by the GBP5 target furin, whereas Syncytin-2 is also efficiently processed by the proprotein convertase subtilisin/kexin type 7 (PCSK7) and thus resistant to GBP5-mediated restriction. Mutational analyses mapped PCSK7 processing of Syncytin-2 to a leucine residue upstream of the polybasic cleavage site. In summary, we identified an innate immune mechanism that impairs the activity of a co-opted endogenous retroviral envelope protein during pregnancy and may potentially contribute to the pathogenesis of pregnancy disorders.

## Teaser

The antiviral activity of GBP5 can be misdirected against co-opted endogenous retroviruses and promote pregnancy complications.

## INTRODUCTION

One important source of genetic innovations is endogenous retroviruses (ERVs). These genomic elements are remnants of retroviruses, which invaded the germ line of our ancestors millions of years ago and continue to do so in some species (*1*). While ERVs have long been regarded as junk DNA, many endogenous retroviral elements have been co-opted during evolution and perform important physiological functions in humans and other vertebrate species. A notable example of exapted ERVs is envelope (*env*) genes that have retained their ability to encode fusogenic glycoproteins and mediate placenta formation in mammals (*2*). For instance, human placenta development depends on the Env proteins Syncytin-1 and Syncytin-2, encoded by the ERVs *ERVW-1* and *ERVFRD-1*, respectively (*3*, *4*). During pregnancy, Syncytin-1 and Syncytin-2 are expressed in placental trophoblast cells. Like typical retroviral Env proteins, Syncytin-1 and Syncytin-2 are glycosylated and proteolytically processed at a polybasic site into surface and transmembrane subunits to become active. The cleavage step is mediated by the ubiquitously expressed serine protease furin and essential for the fusogenic activity of Syncytins (*5*). Once they have reached the plasma membrane, mature trimeric Syncytin-1 and Syncytin-2 interact with their specific receptors ASCT2 or MFSD2A on neighboring cells, respectively (*6*, *7*). Receptor binding triggers the fusion of cytotrophoblast cells and results in the formation of a multinucleated syncytiotrophoblast layer (*8*). This essential component of the human placenta provides a barrier between maternal and fetal bloodstreams and is indispensable for nutrient and gas exchange (*9*–*11*), highlighting the importance of Syncytins for a successful pregnancy outcome. Although Syncytin-1 and Syncytin-2 exert similar functions, they have specialized to some extent. While Syncytin-1 is expressed in cytotrophoblast and syncytiotrophoblast cells, Syncytin-2 expression is largely restricted to the syncytiotrophoblast layer (*12*). In line with this, Syncytin-2 expression increases during spontaneous fusion of primary trophoblast cells in vitro (*13*). This specialization may also explain why Syncytin-1 and Syncytin-2 cannot fully compensate for each other, and why individual depletion of one Syncytin is sufficient to reduce trophoblast fusion (*13*).

While the co-option of retroviral elements represents an elegant source of novel proteins, it may also hamper the distinction between self and nonself by the innate immune system. For example, the usually protective activity of antiviral proteins can be mistargeted against ERVs and interfere with their physiological functions (*14*). One notable example is the inhibition of Syncytin activity by interferon (IFN)–induced transmembrane proteins (IFITMs), which can be aberrantly expressed in the placenta during infections and inflammation, leading to placental dysfunctions and fetal demise in mice (*15*, *16*).

In humans, abnormal Syncytin function and syncytiotrophoblast formation are observed in a variety of pregnancy complications, including the hypertensive disorder preeclampsia (PE) (*17*, *18*) and fetal growth restriction (FGR) (*19*). While the underlying pathomechanisms remain incompletely understood, pregnant women suffering from PE frequently show signatures of immune activation and inflammation (*20*). This aberrant immune activation usually occurs in the absence of any obvious infection and includes elevated levels of cytokines such as CXCL10, IFN-γ, and interleukin-27 (IL-27) and increased expression of antiviral factors (*21*–*25*). We therefore hypothesized that—besides IFITMs—other antiviral proteins targeting viral fusion machineries, such as SERINC5, membrane-associated RING-CH 8 (MARCH8), 90K, and guanylate binding proteins (GBPs), may inhibit Syncytin-mediated trophoblast fusion. Serine incorporator proteins, such as SERINC5, reduce virion infectivity by inhibiting the fusogenic activity of Env (*26*, *27*), while MARCH8 reduces viral glycoprotein levels at the cell surface and prevents furin-mediated proteolytic activation of viral glycoproteins (*28*, *29*). Similarly, GBP2 and GBP5 prevent the maturation of viral Env proteins by inhibiting furin (*28*–*30*). Last, 90K reduces retrovirus infectivity by interfering with the incorporation of Env into nascent virions (*31*).

Here, we show that IFITM3, SERINC5, 90K, MARCH8, GBP2, and GBP5 all inhibit Syncytin-mediated cell fusion. Comparative gene expression profiling revealed that PE and FGR are associated with increased *GBP5* expression in the human placenta. In line with its ability to inhibit furin (*30*), we found that GBP5 reduces the proteolytic cleavage and fusogenic activity of Syncytin-1. In contrast, Syncytin-2 was still efficiently cleaved in the presence of GBP5, and its fusogenic activity was only modestly reduced. Mechanistic analyses revealed that Syncytin-2 can also be activated by the furin paralog PCSK7, which is abundantly expressed in placental trophoblast cells but resistant to GBP5-mediated restriction. In summary, our findings provide a potential mechanistic explanation for impaired syncytiotrophoblast formation in pregnancy complications that are associated with increased expression of GBP5. Furthermore, we show that differences in the mechanisms mediating maturation of Syncytin-1 and Syncytin-2 affect their sensitivity to GBP5-mediated restriction.

## RESULTS

### Inhibition of Syncytin-mediated cell fusion by antiviral proteins

**Fig. 1.**
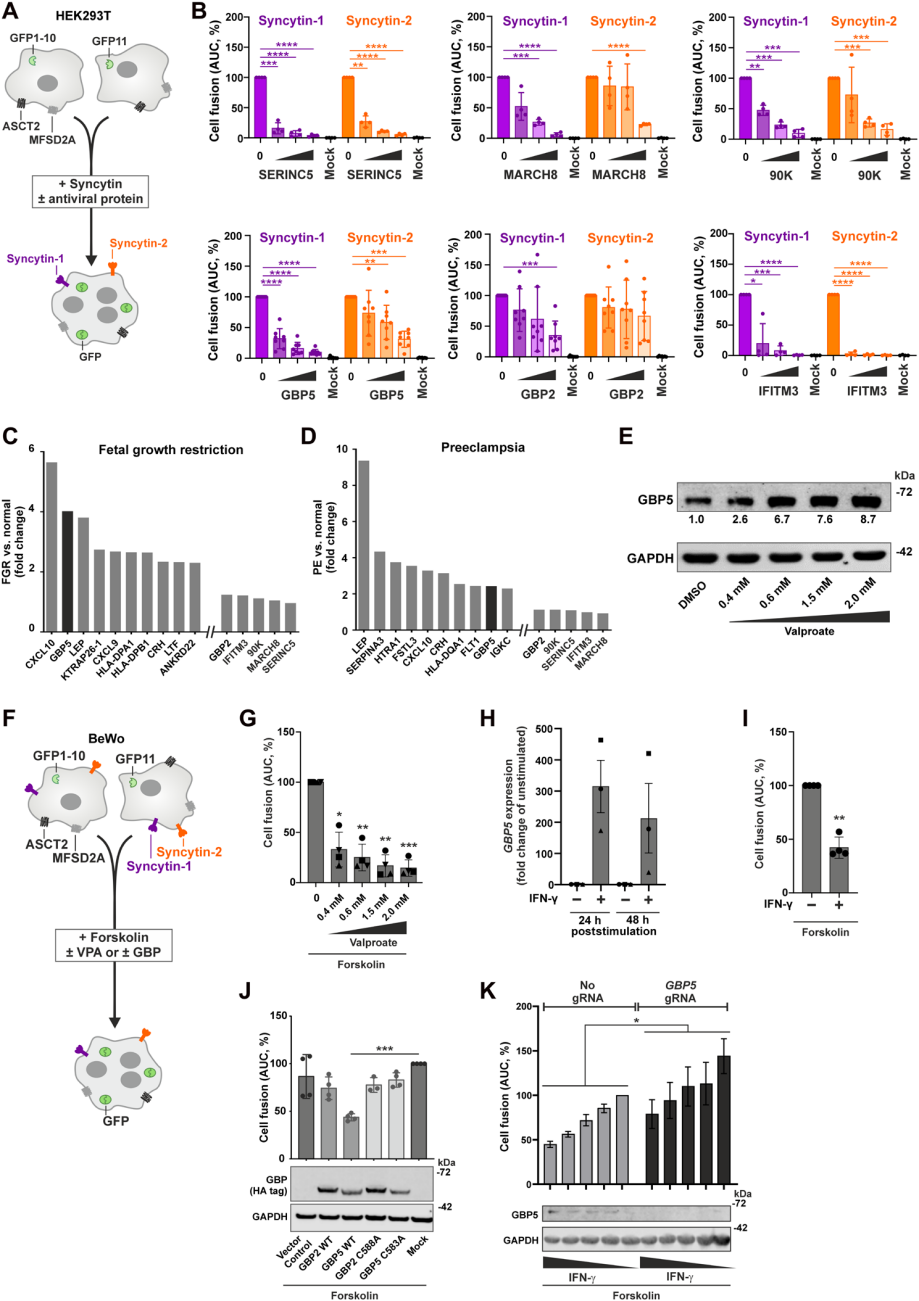
Antiviral proteins inhibit Syncytin-mediated cell fusion. (**A**) HEK293T split-GFP assay. Two subclones of HEK293T cells express one part of GFP each. Upon Syncytin overexpression, the cells fuse and GFP is complemented. (**B**) HEK239T split-GFP cells were cotransfected with Syncytin and antiviral protein. An empty vector was used to adjust total DNA amounts. Fusion was monitored by quantifying GFP fluorescence for 48 hours. Means ± SD, *n* = 4 to 8. (**C** and **D**) Average *n* fold changes of the 10 most strongly up-regulated genes in placentas from patients with FGR or severe PE compared to healthy controls (*n* = 8 per group), and the genes analyzed in Fig. 1B were derived from (*24*). (**E**) GBP5 protein levels in BeWo cells 72 hours after valproate stimulation. (**F**) BeWo split-GFP reporter assay. Upon forskolin stimulation, the cells fuse and GFP is complemented. (**G**) BeWo cell fusion upon valproate stimulation. Means ± SD, *n* = 4. (**H**) *GBP5* expression upon IFN-γ stimulation (100 ng/ml) in BeWo cells, quantified by quantitative PCR. Means ± SEM, *n* = 3. (**I**) BeWo cell fusion upon IFN-γ stimulation (100 ng/ml). Means ± SD, *n* = 4. (**J**) BeWo cell fusion after *GBP2/5* overexpression. BeWo split-GFP cells were transduced with GBP2/5 and treated with forskolin, and GFP fluorescence was monitored for 72 hours. Means ± SD, *n* = 3 to 4. (**K**) Cell fusion after *GBP5* depletion. BeWo split-GFP cells were transduced with CRISPR/Cas lentiviruses expressing *GBP5*-targeting single guide RNA (sgRNA) or a negative control. Three days later, cells were stimulated with IFN-γ, before fusion was induced by forskolin 2 days later. GFP fluorescence was monitored for 44 hours. Means ± SEM are shown on top, *n* = 3. One representative Western blot is shown at the bottom. A one-way analysis of variance (ANOVA) with multiple comparisons (Dunnett’s test) was performed for (G), (J), and (K). A paired *t* test was performed for (I) (*P < 0.05, **P < 0.01, ***P < 0.001, and ****P < 0.0001). AUC, area under the curve; DMSO, dimethyl sulfoxide; h, hours.

To monitor Syncytin-mediated cell fusion in the presence or absence of antiviral proteins that target the viral fusion machinery, we used a split–green fluorescent protein (GFP) system (*15*). Here, two independent cell populations express half of the reporter protein that complement each other and generate a fluorescence signal upon cell fusion (Fig. 1A). Notably, human embryonic kidney (HEK) 293T cells endogenously express ASCT2 and MFSD2A (*32*), the receptors of Syncytin-1 and Syncytin-2, respectively (*6*, *7*, *33*). Consequently, exogenous expression of Syncytin-1 or Syncytin-2 is sufficient to trigger cell fusion and thus GFP complementation. We found that SERINC5, MARCH8, 90K, GBP5, and—to a lesser extent—GBP2 suppress Syncytin-1– and Syncytin-2–mediated membrane fusion (Fig. 1B and fig. S1). IFITM3 served as positive control since this antiviral protein was previously shown to reduce the fusogenic activity of both Syncytins, even more efficiently than its paralogs IFITM1 and IFITM2 (*15*, *16*). In some cases, e.g., MARCH8 and GBP5, Syncytin-2 was less sensitive to restriction compared to Syncytin-1. Together, Syncytin-mediated cell fusion is inhibited by several antiviral effectors that target retroviral envelope proteins.

### Association of pregnancy complications with increased *GBP5* expression

To investigate a potential pathophysiological relevance of the antiviral proteins in pregnancy complications, we took advantage of the publicly available transcriptomic data of primary placenta samples. We focused on patients suffering from FGR or PE since these disorders are known to be associated with impaired syncytiotrophoblast formation and aberrant placenta development (*18*). A comparative gene expression profiling by Nishizawa and colleagues (*24*) revealed that *GBP5* ranks second and ninth among genes that are up-regulated in placentas from patients with FGR and PE, respectively, compared to placentas of healthy women (Fig. 1, C and D). Although this increase was statistically not significant, it distinguished *GBP5* from *GBP2*, *90K*, *MARCH8*, *SERINC5*, and *IFITM3*, whose expression was not elevated in placentas from patients with PE or FGR (Fig. 1, C and D). Of note, a similar expression pattern was observed for in vitro cultured cytotrophoblast cells isolated from PE or control placentas (*34*). Again, *GBP5* expression was increased about two- to fourfold in PE versus control samples (fig. S2). Last, increased *GBP5* expression was found to be also associated with unexplained preterm birth (*35*, *36*). In line with this, *GBP5* mRNA levels were increased in a preterm labor mouse model (*37*).

Impaired placental function and reduced trophoblast cell fusion are also observed in response to the antiepileptic drug valproate (*38*, *39*). This histone deacetylase (HDAC) inhibitor is contra-indicated during pregnancy as it can cause a range of developmental delays and congenital malformation that are referred to as fetal valproate spectrum disorder (FVSD) or fetal valproate syndrome (*40*). Since *GBP5* expression is driven by an endogenous retroviral promoter that is activated upon HDAC inhibition (*41*–*43*), we hypothesized that valproate may induce its expression in placental cells. Valproate dose-dependently increased GBP5 protein levels in BeWo cells, a well-characterized trophoblast cell line (Fig. 1E) (*44*). To investigate whether valproate affects fusion of placental cells, we took advantage of BeWo cells stably expressing the split-GFP reporter proteins (*15*). Syncytia formation of BeWo cells can be triggered by forskolin stimulation and involves both, Syncytin-1– and Syncytin-2–mediated cell fusion (Fig. 1F) (*13*). Upon treatment of cells with increasing amounts of valproate, we observed a dose-dependent inhibition of forskolin-triggered fusion of BeWo cells (Fig. 1G and fig. S3, A and B). Since IFN-γ is a strong inducer of *GBP5* (*30*) and increased in plasma of pregnant women suffering from PE (*21*), we hypothesized that it may also interfere with Syncytin-mediated cell fusion. IFN-γ induced GBP5 expression in BeWo cells (Fig. 1H) and significantly reduced forskolin-induced syncytia formation (Fig. 1I).

Last, we analyzed the effect of *GBP* overexpression and depletion on fusion of placental BeWo cells. Expression of wild-type (WT) GBP5 reduced trophoblast cell fusion by around 50%. GBP2 and two previously characterized GBP mutants (GBP2-C588A and GBP5-C583A) that lack their C-terminal isoprenylation motifs and lost their ability to restrict viral glycoproteins (*30*) showed no significant inhibition of Syncytin-mediated cell fusion (Fig. 1J and fig. S3C). Depletion of GBP5 had the opposite effect and increased BeWo cell fusion by about 50% (Fig. 1K).

In summary, elevated *GBP5* expression is associated with PE, FGR, and FVSD. Furthermore, the fusion of trophoblast cells is enhanced upon knockout of *GBP5* but inhibited upon GBP5 overexpression, as well as stimulation with valproate and IFN-γ, both of which also induce *GBP5* expression. Because of these associations between pregnancy-related disorders and increased *GBP5* expression, we focused our further analyses on the interplay of GBP5 with Syncytin function and trophoblast cell fusion.

### GBP5-mediated suppression of Syncytin-1 but not Syncytin-2 cleavage

**Fig. 2.**
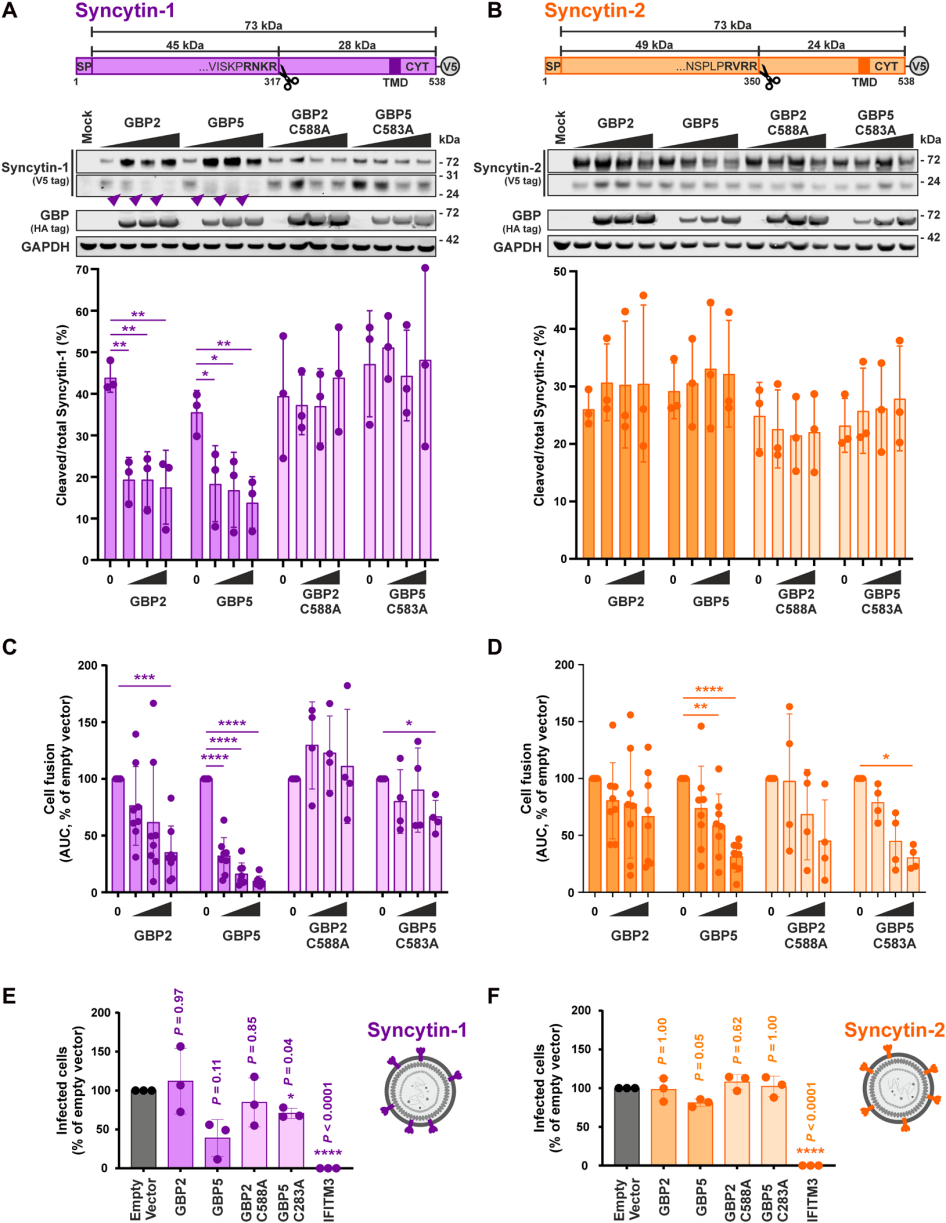
GBP2 and GBP5 inhibit the proteolytic activation of Syncytin-1, but not Syncytin-2. (**A** and **B**) Syncytin cleavage in the presence of GBPs. Top illustrates the topologies of Syncytin-1 and Syncytin-2, including their polybasic cleavage sites. To monitor Syncytin cleavage, HEK293T cells were cotransfected with Syncytin expression plasmids and increasing amounts of GBP expression plasmids. Two days posttransfection, cells were harvested, and cleavage was analyzed by Western blotting. Syncytin maturation was determined by calculating the amount of cleaved to total Syncytin. Representative Western blots are shown in the middle. Purple arrowheads indicate the shift in the electrophoretic mobility of Syncytin-1. Bottom shows quantifications of three independent Western blots ± SD. SP, signal peptide; TMD, transmembrane domain; CYT, cytoplasmic tail; V5, V5 tag. (**C** and **D**) Syncytin-mediated cell fusion in the presence of GBPs. HEK239T cells stably expressing the N-terminal portion of GFP were cotransfected with expression plasmids for Syncytin-1 or Syncytin-2 and increasing amounts of plasmids expressing GBP2 (C) or GBP5 (D) and mixed with HEK293T cells expressing C-terminal part of GFP. GFP fluorescence was quantified over a period of 48 hours as a marker for syncytia formation. Mean values of four to eight independent experiments ± SD are shown. (**E** and **F**) Infectivity of Syncytin-carrying pseudovirions produced in the presence of GBPs. *MFSD2A* knockout HEK293T cells were cotransfected with plasmids for Syncytin-1 or Syncytin-2 together with ASLV *gag*-*pol*, mScarlet minigenome, and GBP or IFITM3. Two days later, supernatants were collected and used for the infection of HEK293T cells overexpressing *MFSD2A*. Three days postinfection, the percentage of infected cells was analyzed by flow cytometry. IFITM3 served as a positive control. Mean values of three independent experiments ± SD are shown. A one-way ANOVA with multiple comparisons (Dunnett’s test) was performed (*P < 0.05, **P < 0.01, ***P < 0.001, and ****P < 0.0001).

We previously showed that GBP5 and—to a lesser extent—GBP2 suppress the proteolytic maturation of viral envelope proteins by inhibiting the cellular protease furin (*30*). Since furin has been shown to proteolytically activate Syncytins at polybasic cleavage sites (*5*), we hypothesized that increased *GBP2/GBP5* expression in the placenta may reduce the maturation and fusogenic activity of both Syncytins. Overexpression of *GBP2* and *GBP5* reduced the proteolytic cleavage of Syncytin-1 into its mature form (Fig. 2A). In contrast, the isoprenylation mutants, GBP2-C588A and GBP5-C583A, did not interfere with the proteolytic maturation of Syncytin-1. We also observed a shift in the electrophoretic mobility of cleaved Syncytin-1 in the presence of WT GBP2 and GBP5 (Fig. 2A). This shift was lost upon treatment with peptide:*N*-glycanase F (PNGase F) (fig. S4A), indicating that GBP2 and GBP5 interfere with N-linked glycosylation of Syncytin-1, as previously described for other retroviral glycoproteins (*30*, *42*, *45*). Notably, analysis of PNGase F–treated samples confirmed reduced cleavage of Syncytin-1 in the presence of GBP2 and GBP5 (fig. S4A). Unexpectedly, Syncytin-2 was still efficiently cleaved in the presence of these two antiviral proteins (Fig. 2B).

To investigate whether furin inhibition is required for the ability of GBPs to suppress Syncytin-mediated fusion, we analyzed WT and mutant GBP2 and GBP5 side by side in the split-GFP HEK293T fusion assay described above (Fig. 1A). While WT GBPs efficiently inhibited Syncytin-1–mediated cell fusion, the isoprenylation mutants had no marked effect (Fig. 2C). In contrast, there was no significant difference between WT and mutant GBPs in case of Syncytin-2 (Fig. 2D), suggesting that Syncytin-2–mediated cell fusion may be inhibited by GBP5 via an alternative pathway.

Syncytins are derived from Env proteins that were integral parts of viral particles, where they mediated receptor binding and ultimately entry of the virion into the target cells. To validate the impact of GBPs on the activity of Syncytins in independent experimental setups, we generated viral particles harboring Syncytin-1 or Syncytin-2 and monitored their infectivity in the presence of GBPs. In our first approach, we pseudotyped a GFP-expressing vesicular stomatitis virus (VSV) with Syncytins (fig. S4B). In agreement with the cell fusion assays (Fig. 2, C and D), the infectious yield of Syncytin-1–pseudotyped particles was reduced in the presence of WT GBP2 and GBP5, whereas the isoprenylation mutants had no marked effect (fig. S4, B and C). Since Syncytin-2–pseudotyped VSV particles were not infectious, we also pseudotyped an avian retrovirus with Syncytin-1 or Syncytin-2 and infected HEK293T cells with or without the Syncytin-1 and Syncytin-2 receptors ASCT2 or MFSD2A, respectively (Fig. 2, E and F, and fig. S5, A and B). Syncytin-1 pseudovirions infectivity was inhibited by WT GBP5, but not GBP2, compared to an empty vector control. In contrast, Syncytin-2 virions were largely resistant to both GBPs but sensitive to IFITM3-mediated restriction (Fig. 2, E and F, and fig. S5B), although GBP protein levels were similar in cell producing Syncytin-1 or Syncytin-2 virions (fig. S5C).

Together, these data show that GBP5 reduces the proteolytic cleavage and fusogenic activity of Syncytin-1, while Syncytin-2–mediated fusion is inhibited via an alternative mechanism independently of altered Syncytin-2 cleavage.

### Efficient cleavage of Syncytin-2 by PCSK7

**Fig. 3.**
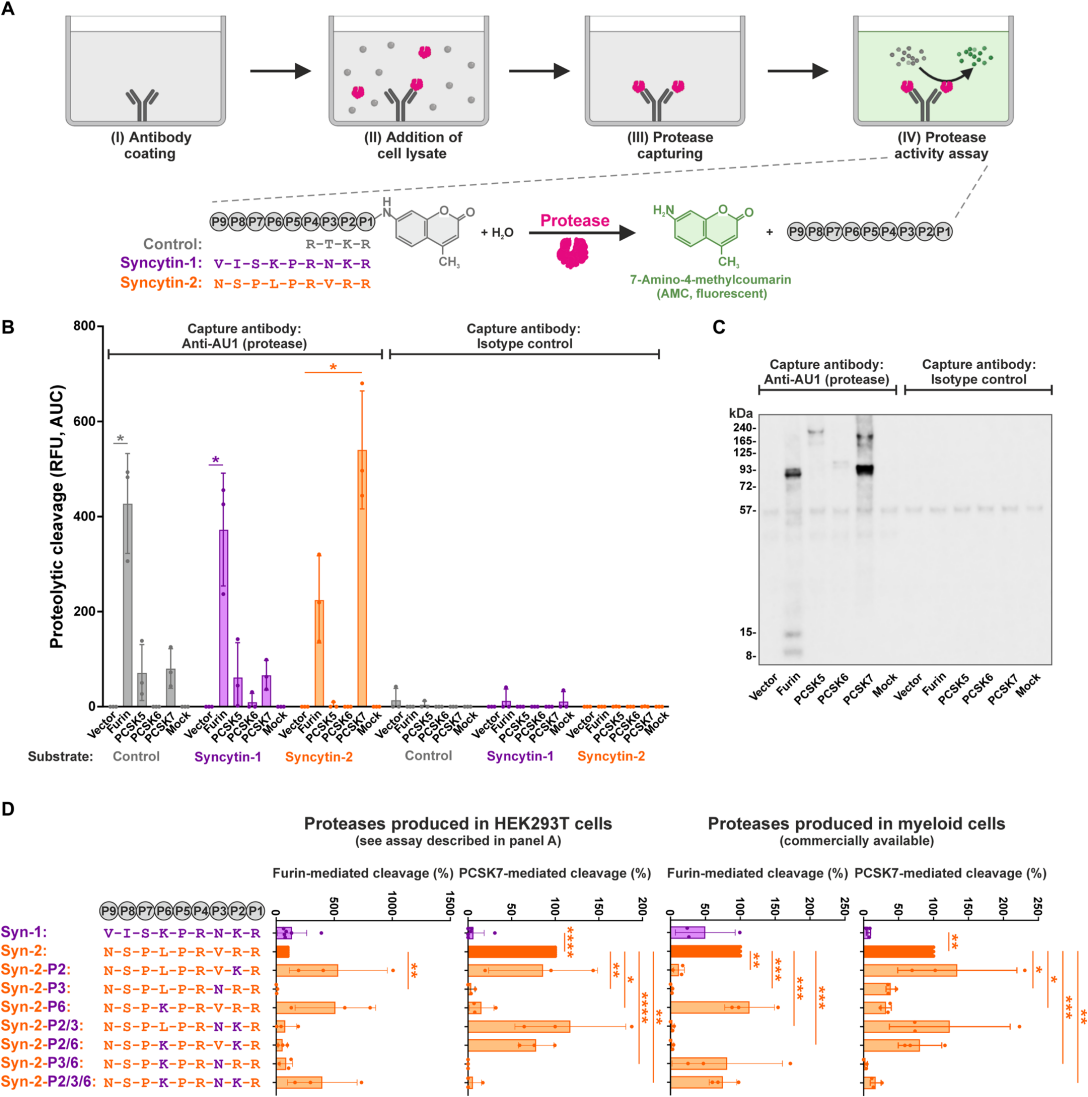
Syncytin-2 is cleaved by PCSK7. (**A**) Principle of the PCSK capture assay. (I) A 96-well plate is coated with antibodies against the AU1 tag or the respective isotype control. (II) Lysates of HEK293T cells overexpressing individual, AU1-tagged PCSKs are added, and (III) proteases are captured and washed. (IV) Captured proteases are incubated with AMC reporter substrates harboring the cleavage sites of Syncytin-1, Syncytin-2, or a furin consensus cleavage site, and fluorescence signal is integrated with a plate reader. (**B**) PCSK-mediated cleavage of Syncytins. The ability of furin, PCSK5, PCSK6, and PCSK7 to cleave Syncytin-1 or Syncytin 2 was determined as described in (A). Mean values of three independent experiments ± SD are shown. A paired *t* test was performed (*P < 0.05). RFU, relative fluorescence units. (**C**) Western blot analysis of captured proteases. Capture efficiency of PCSKs was monitored by Western blotting. One exemplary Western blot of the experiments shown in (B) is shown. (**D**) Furin- and PCSK7-mediated cleavage of mutated Syncytin substrates. AMC reporter substrates harboring the cleavage sites (P1 to P9) of Syncytin-1 or Syncytin-2 are shown on the top left. The chimeras that were generated to map determinants of PCSK7 cleavage are shown below. Amino acids found in Syncytin-1 and Syncytin-2 are shown in purple and orange, respectively. The AMC reporter substrates shown on the left were either incubated with proteases captured from transfected HEK293T cells as described in Fig. 3A (middle) or commercially available proteases produced in myeloid cells (right). Substrate cleavage was monitored over time, and the area under the curve was calculated. Mean values of three to six independent experiments ± SD are shown. A one-way ANOVA with multiple comparisons (Dunnett’s test) was performed (*P < 0.05, **P < 0.01, ***P < 0.001, and ****P < 0.0001).

Since proteolytic cleavage of Syncytin-2 was not affected by GBP2 and GBP5 (Fig. 2B), we hypothesized that Syncytin-2 can be activated not only by furin but also by other proteases. Likely candidates are the pro–protein convertases (PCs) PCSK1, PCSK2, PCSK4, PCSK5, PCSK6, and PCSK7. Like furin (which is also known as PCSK3), they recognize and cleave polybasic motifs with the consensus sequence RX[K/R]R (*46*). Publicly available single-cell RNA sequencing of healthy term placentas revealed that furin, as well as PCSK5, PCSK6, and PCSK7 are expressed in trophoblast cells, while PCSK1, 2, and 4 are not (figs. S6 and S7, upper part) (*12*). We therefore directly compared the processing of the polybasic Syncytin-1 and Syncytin-2 cleavage sites by furin, PCSK5, PCSK6, and PCSK7. To do so, these four proteases were isolated from lysates of transfected HEK293T cells, and their activity was determined using a 7-amido-4-methylcoumarin (AMC) reporter assay (Fig. 3A). In this assay, a substrate harboring the cleavage sites of Syncytin-1 (VISKPRNKR), Syncytin-2 (NSPLPRVRR), or a minimal PCSK consensus cleavage site (RTKR) is processed into a fluorescent product (Fig. 3A). As expected, furin cleaved all three substrates with similar efficacy (Fig. 3B). In contrast, PCSK5 and PCSK6 showed generally low substrate cleavage, most likely as a result of lower total cellular (fig. S8A) and purified, captured (Fig. 3C) protein levels. Last, PCSK7 efficiently cleaved the Syncytin-2, but not Syncytin-1 substrate (Fig. 3B), suggesting that this protease, which is abundantly expressed in the placenta (figs. S6 and S7), may mediate proteolytic activation of Syncytin-2. In line with these findings, commercially available PCSK7 purified from myeloma cells efficiently cleaved the Syncytin-2 substrate, while furin was more efficient in cleaving Syncytin-1 (fig. S8B). Together, while furin is able to cleave both Syncytins, Syncytin-2 is additionally processed by PCSK7.

### Determinants of Syncytin-2 cleavage by PCSK7

To identify potential determinants of PCSK7 cleavage in Syncytin-2, we compared the cleavage sites of Syncytin-1 and Syncytin-2 (Fig. 3D). Within the polybasic core motif (RX[K/R]R), the two proteins differ in the residues at P2 and P3, which are lysine and asparagine in Syncytin-1, but arginine and valine in Syncytin-2. Notably, PCSK7 substrates were shown to be enriched for leucine residues upstream of the cleavage site (*47*), while furin prefers basic residues at positions P5 and P6 (*48*). In line with this, Syncytin-2 harbors a leucine at P6, while Syncytin-1 carries a lysine (Fig. 3D). We therefore determined the role of P2, P3, and P6 in PCSK7-mediated cleavage of Syncytin-2 by exchanging the respective amino acids to those found in Syncytin 1, either alone or in combination (Fig. 3D). The AMC reporter assay revealed that exchange of the P2 residue (arginine to lysine) did not significantly affect cleavage of Syncytin-2 by PCSK7. This was true for both, proteases captured from the lysates of the transfected HEK293T cells (Fig. 3D left, and fig. S8C) and commercially available proteases produced in myeloid cells (Fig. 3D, right). In contrast, mutation at position P3 abrogated substrate cleavage by both, furin and PCSK7. Last, introduction of a lysine residue at position P6 of Syncytin-2 significantly reduced its processing by PCSK7 while increasing cleavage by furin (Fig. 3D). Thus, P6 in the polybasic cleavage site is a key determinant of PCSK7- versus furin-mediated processing of Syncytins.

### Resistance of PCSK7 to GBP2/5-mediated restriction

**Fig. 4.**
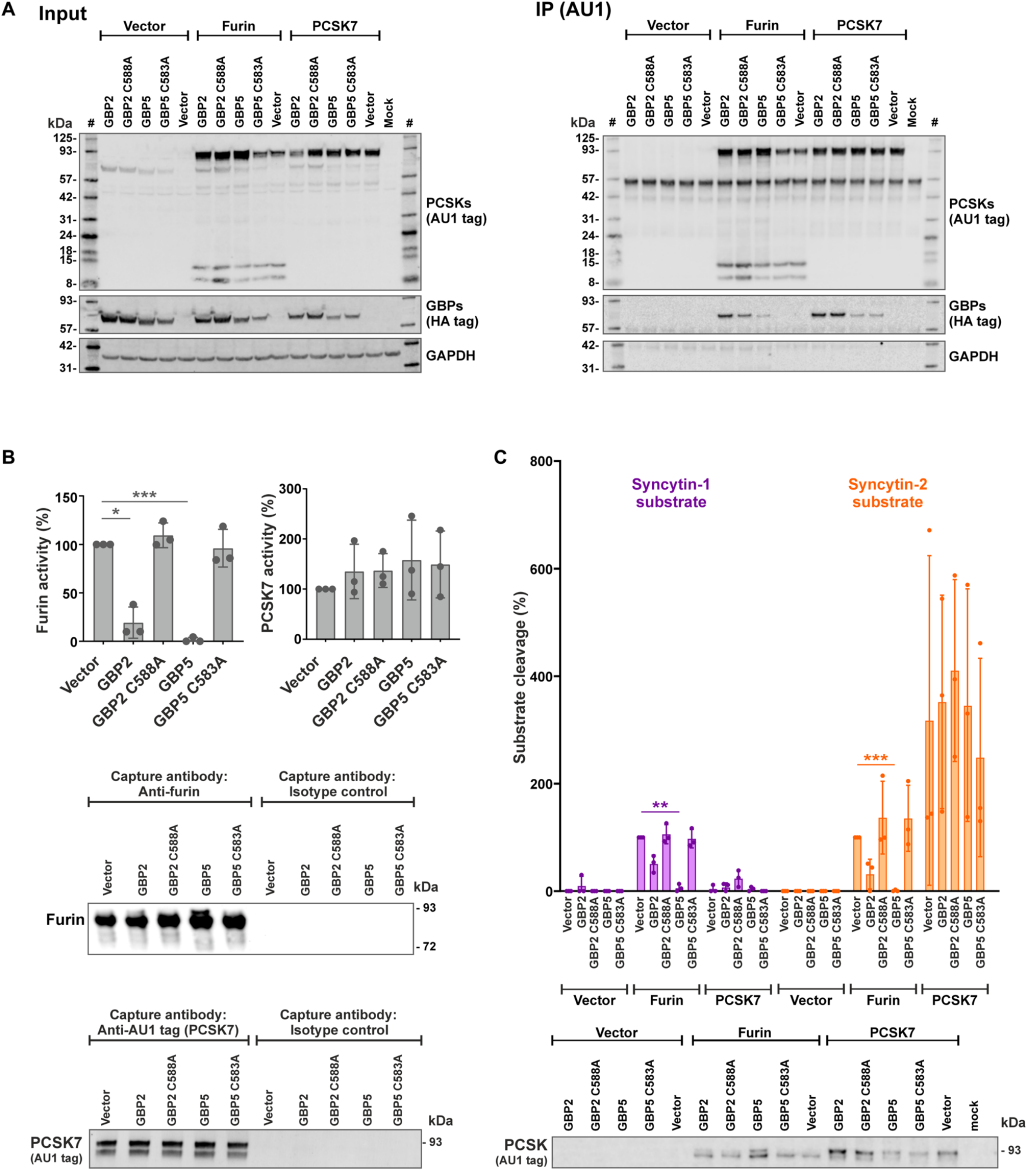
PCSK7 is resistant to GBP-mediated restriction. (**A**) Interaction of furin or PCSK7 with GBPs. HEK293T cells were cotransfected with expression plasmids for the indicated PCSKs and GBPs or the respective vector controls. One day posttransfection, cells were harvested, and furin or PCSK7 were pulled down via their C-terminal AU1 tag. Western blotting was performed to detect PCSKs and GBPs in whole cell lysates (left) and the pull down (right). The results of one of three independent experiments are shown. IP, immunoprecipitation; #, protein size marker. (**B** and **C**) Furin- and PCSK7-mediated Syncytin cleavage in the presence of GBPs. Furin and PCSK7 were captured from cell lysates of transfected HEK293T cells essentially as described in Fig. 3A. However, cells were additionally cotransfected with expression plasmids for GBP2 or GBP5 (WT or isoprenlyation mutant) or the respective vector control. The ability of captured furin/PCSK7 to process a consensus furin cleavage site (B) or the Syncytin-1 and Syncytin-2 cleavage sites (C) were analyzed using the AMC reporter assay described in Fig. 3A. Mean values of three independent experiments ± SD are shown on top. A one-way ANOVA with multiple comparisons (Dunnett’s test) was performed (*P < 0.05, **P < 0.01, and ***P < 0.001). Capture efficiency of PCSKs was monitored by Western blotting and is shown at the bottom.

Since Syncytin-2 can be cleaved and activated by PCSK7 (Fig. 3 and fig. S8B) and Syncytin-2 cleavage is resistant to GBP2/5 (Fig. 2B), we directly compared the effects of GBP2/5 on furin and PCSK7. Coimmunoprecipitation experiments in HEK293T cells cotransfected with furin/PCSK7 and WT or mutant GBPs showed that both, furin and PCSK7, pulled down WT GBP2 and GBP5 (Fig. 4A). As expected, furin captured from cells expressing GBP2 or GBP5 lost its ability to cleave the RTKR reporter substrate in the AMC reporter assay (Fig. 4B, upper part), although the levels of captured protease were similar in all experimental conditions (Fig. 4B, lower part). In contrast, PCSK7-mediated cleavage was not affected by GBP2 or GBP5 (Fig. 4B). In agreement with these findings, furin produced in the presence of GBPs also lost its ability to process the Syncytin-1 and Syncytin-2 cleavage sites (Fig. 4C, upper part) despite similar protease capture efficiencies (Fig. 4C, lower part). However, Syncytin-2 cleavage by PCSK7 was not altered in the presence of GBPs (Fig. 4C). Together, Syncytin-1 is primarily cleaved by furin, whose activity is inhibited by GBP2/5, while Syncytin-2 is efficiently processed by PCSK7, which is not affected by GBP2 or GBP5.

## DISCUSSION

GBP5 is a well-characterized antiviral protein targeting the maturation and fusogenic activity of diverse viral glycoproteins (*30*, *45*, *49*). Here, we show that GBP5 also prevents the activation and fusogenic activity of the endogenous retroviral protein Syncytin-1. Although a direct connection remains to be established, our findings provide a possible mechanistic link between increased GBP5 expression in trophoblast cells and impaired placenta formation. Usually, Syncytins are proteolytically activated by furin and mediate fusion of cytotrophoblast cells into the syncytiotrophoblast layer, an essential step during human placenta formation (*3*, *4*). However, the ability of Syncytin-1 and—to a lesser extent—Syncytin-2 to mediate cell fusion is impaired in the presence of GBP5 (Fig. 1, B, J, and K). The reduced sensitivity of Syncytin-2 could be ascribed to the P6 residue of its cleavage site. This site enables alternative proteolytic activation of Syncytin-2 by PCSK7, a paralog of furin that is resistant to GBP5-mediated restriction.

In line with a detrimental role of GBP5 during placenta development, primary trophoblast cells from healthy pregnancies do not express detectable levels of *GBP5* (fig. S7) (*12*). In contrast, global expression analyses revealed that induction of *GBP5* in placental cells is associated with pregnancy complications such as PE, suggesting a pathophysiological relevance of this factor in vivo (Fig. 1, C and D, and fig. S2) (*35*–*37*). PE is a multisystem disorder that affects about 2 to 8% of all pregnancies (*50*). It is characterized by impaired syncytiotrophoblast formation, hypertension, and organ manifestations such as proteinuria (*50*). If left untreated, then it can result in seizures and increase the risk of lethal outcomes for both the mother and the fetus (*50*). Of note, PE in humans is associated not only with increased *GBP5* expression but also with decreased furin levels in plasma (*51*). It is tempting to speculate that reduced furin plasma levels are a result of the ability of GBP5 to inhibit the secretion of this protease (*30*).

Pregnant women suffering from PE typically also show signs of inflammation, despite the absence of any obvious infection. For example, cytokines such as IFN-γ and IL-27 are increased in patients with PE (*21*, *25*, *52*, *53*). These cytokines may contribute to the detrimental expression of *GBP5* in the placenta since this antiviral gene is strongly induced upon IFN-γ and IL-27 stimulation (*30*, *54*). Similarly, Buchrieser and colleagues (*15*) proposed that IFN-mediated induction of IFITM proteins during viral infections may prevent normal placenta development and potentially result in fetal demise. In contrast to GBP5, however, which is primarily induced by type II IFNs, they reported that IFITMs are mainly induced by type I IFNs that are increased in response to infection (*15*). In the context of PE, however, type I IFNs or IFITMs are not elevated (Fig. 1D).

The expression of *GBP5* is regulated by an endogenous retroviral LTR12C promoter, which was inserted directly upstream of the *GBP5* coding sequence during primate evolution (*42*). This insertion most likely also led to the gain of IFN-γ responsiveness of *GBP5*. Upon infections with furin-dependent viruses such as HIV, this may provide a selection advantage as it increases the expression of *GBP5*, thereby impairing furin-mediated activation of viral glycoproteins. During pregnancy, however, the IFN-γ responsiveness of *GBP5* may come at the cost of impaired Syncytin-1 activation and aberrant placenta formation. PE seems to be a pregnancy disorder that is unique to humans and related great ape species (*42*, *55*), which harbor the LTR12C promoter upstream of *GBP5* (*42*). In contrast, PE is not observed in monkeys or other mammals that lack the LTR12C insertion (*42*, *55*). LTR12C promoters are also well-known for their responsiveness to HDAC inhibition (*41*, *56*). In agreement with this, we found that the HDAC inhibitor valproate enhances *GBP5* expression in placental BeWo cells and reduces trophoblast fusion (Fig. 1, E to G, and fig. S3, A and B) (*38*). Thus, our findings suggest that valproate-induced *GBP5* expression may contribute to reduced cytotrophoblast cell fusion and ultimately promote the pathogenesis of the fetal valproate syndrome observed in pregnant women treated with this anti-epileptic drug.

One limitation of our study is the lack of an animal model that enables us to validate a direct pathophysiological link between *GBP5* expression and pregnancy complications such as PE. At first glance, *GBP5* knockout mouse models may seem helpful. However, the interplay of GBP5, Syncytins, and human placenta development is unique in several aspects: Syncytin-1 and Syncytin-2 are only found in primates, and PE is most likely unique to humans and some related great ape species (*55*). While small animals, such as mice, also use retroviral Env proteins to mediate placenta cell fusion (*2*), these Syncytins are of distinct origin. Moreover, the *GBP* gene locus experienced multiple gene duplication and loss events during mammalian evolution and is only poorly conserved between mice and humans (*57*, *58*). Last, the responsiveness of *GBP5* to cytokine stimulation is species specific due the above-mentioned insertion of a retroviral LTR12C promoter (*42*).

Apart from a mechanistic link between increased *GBP5* expression and impaired trophoblast cell fusion, our study provides key insights into the proteolytic maturation and protease dependencies of Syncytins. While the furin inhibitor GBP5 interfered with the proteolytic activation of Syncytin-1, it had no effect on the cleavage of Syncytin-2 (Fig. 2, A and B). This came as a surprise since both Syncytins can be processed by furin (*5*). A comparison of different PCSKs expressed in healthy primary placental cells (fig. S7) revealed that the polybasic cleavage site of Syncytin-2 can also be efficiently processed by the furin paralog PCSK7. However, efficient cleavage of full-length Syncytin-2 by PCSK7 remains to be demonstrated. While we did not formally test this, cleavage by PCSK7 most likely activates Syncytin-2, since furin and PCSK7 both cleave their target proteins at identical positions, i.e., downstream of the RX[K/R]R core motif. Notably, our findings also help to explain earlier, seemingly discrepant results in placenta research. While the furin inhibitor Dec-RVKR-CMK suppressed trophoblast cell fusion, specific knockout of furin did not (*59*). Our results strongly suggest that PCSK7 enabled Syncytin-mediated trophoblast fusion upon knockout of furin in these experiments but failed to do so in the presence of Dec-RVKR-CMK, since this inhibitor targets several PCSKs, including PCSK7 (*60*). This observation also illustrates the difficulty of developing specific PCSK inhibitors since PCSK1-7 recognize a similar polybasic target sequence. Our comparison of Syncytin-1 versus Synctin-2 cleavage helps to overcome this hurdle by identifying determinants of protease/substrate specificity. Our mutational analyses revealed that a leucine residue at position P6 enables efficient cleavage of Syncytin-2 by PCSK7 (Fig. 3). Mutation of this residue to lysine reduced cleavage of Syncytin-2 by PCSK7 while simultaneously increasing furin-mediated processing.

The inhibition of Syncytin-1 cleavage by GBP5 explains why its fusogenic activity is reduced in the presence of this antiviral protein. However, although Syncytin-2 was efficiently cleaved in the presence of GBP5 (Fig. 2A), its ability to mediate syncytia formation was still reduced, albeit to a lesser extent than that of Syncytin-1 (Fig. 1B). This inhibitory effect was also observed for the isoprenylation-deficient mutant of GBP5 (Fig. 2D), demonstrating that it is independent of the inhibition of furin. In a recent publication, Wang and colleagues propose that GBP5 not only targets furin but also binds to the oligosaccharyltransferase complex, thereby inhibiting N-linked glycosylation and proper maturation of diverse viral glycoproteins (*61*, *62*). In agreement with these findings, we observed altered N-linked glycosylation of Syncytin-1 in the presence of GBP5 (fig. S4A). However, we did not observe any obvious changes in the electrophoretic mobility of Syncytin-2 that may indicate altered glycosylation. Thus, the exact role of the oligosaccharyltransferase complex in GBP5-mediated Syncytin maturation remains to be determined.

In conclusion, our finding that GBP5 impairs the maturation and function of Syncytin-1 provides a plausible explanation for the association of increased *GBP5* expression with PE, FGR, and the fetal valproate syndrome. Pathological mistargeting of Syncytin-1 by GBP5 also illustrates that the co-option of endogenous retroviral genes may come at the cost of impaired discrimination between self and nonself by our immune system.

## MATERIALS AND METHODS

### Cell culture

The human HEK293T (American Type Culture Collection, catalog no. CRL-3216), HEK293T split-GFP 1-10, and HEK293T split-GFP 11 cells (*15*) were grown in Dulbecco’s modified Eagle’s medium (DMEM) medium supplemented with 10% fetal calf serum (FCS), streptomycin (100 μg/ml), and penicillin (100 U/ml). BeWo split-GFP 1-10 and BeWo split-GFP 11 cells were grown in DMEM medium supplemented with 20% FCS, streptomycin (100 μg/ml), and penicillin (100 U/ml). ASCT1/2 double knockout and *MFSD2A* knockout clones were grown in DMEM medium supplemented with 5% FCS, 5% calf serum, streptomycin (100 μg/ml), and penicillin (100 U/ml). All cells were cultured under a 5% CO_2_ atmosphere at 37°C and 90% relative humidity.

The ASCT1/2 CRISPR-mediated double knockout clone 3F9-2E12 (FE) was derived from the HEK293T cell line as described before (*33*). The MFSD2A knockout clone C89C1 was prepared similarly by targeting the *MFSD2A* gene with guide RNA 5′AAGCUACUGUAGAGCUAUUG. The C89C1 clone carries a heterozygous frameshift mutation in exon 6 (fig. S5A), which was verified by Sanger and nanopore sequencing.

### Plasmids and cloning

The generation of pCG vectors expressing N-terminally hemagglutinin (HA)–tagged GBP2 or GBP5 (WT and isoprenylation-deficient mutants) has been described before (*30*). Briefly, the GBP coding sequence was inserted via Xba I/Mlu I. The expression plasmid for HA-tagged IFITM3 was cloned in the same way. The generation of the pCAGGS vector expressing N-terminally tagged MARCH8 has been described by Tada and colleagues (*28*). In this case, the MARCH8 coding sequence was inserted via Xho I/Not I. pcDNA6 expressing myc-tagged 90K has been generated by Lodermeyer *et al*. (*31*). SERINC5 was polymerase chain reaction (PCR)–amplified from already existing plasmid using Phusion High Fidelity DNA polymerase (Thermo Fisher Scientific). The pCG vector was digested with Xba I and Mlu I restriction enzymes from New England Biolabs (NEB). The PCR product and digested vector were gel-purified using the Monarch DNA Gel Extraction Kit (NEB). Last, the gene was inserted in the vector using In-Fusion cloning (Takara) according to the manufacturer’s protocol. The pCMV plasmids expressing untagged Syncytin-1 and Syncytin-2 have been described before (*15*). A C-terminal V5 tag was added. Furin was C-terminally AU1-tagged and expressed from pCG expression plasmids. The generation of this plasmid via Xba I/Mlu I digestion and ligation was described before (*30*). Expression plasmids encoding C-terminally AU1-tagged PCSK5, PCSK6, and PCSK7 were generated in the same way. The coding sequence of MFSD2A (NP_116182.2) was cloned into a previously described gamma-retroviral vector and fused to the coding sequence of GFP (*33*).

pMD2.G, pMDLg/pRRE, and pRSV *rev* have been described before (*63*) and were obtained through addgene (catalog nos. 12259, 12251, and 12253) to generate third-generation lentiviruses. For lentiviral transduction, pCSC-SP-PW-GFP (pBOB-GFP) from addgene (catalog no. 12337) (*64*) was modified to express a blasticidin resistance gene via an internal ribosomal entry site instead of *GFP*. *GBP2* and *GBP5* (WT or isoprenylation mutants) were inserted into pCSC-SP-PW via Xba I/Bam HI. An N-terminal HA tag was added.

For the generation of alpharetrovirus pseudotypes, Syncytin-1 with a truncated cytoplasmic domain (stop codon after leucine 485) was expressed from a plasmid described previously (*33*). The coding sequence of Syncytin-2 (NP_997465.1) was cloned into the same expression vector, also harboring a truncated cytoplasmic domain (stop codon after leucine 520). Production of pseudotyped virions was ensured by human codon–optimized alpharetroviral *gag* and *pol* (*65*). pAlpha-SF-mScalret-wPRE derived from pAlpha-SF-mCherry-wPRE was used as the transduction marker genome (*33*) by exchange of the gene encoding the fluorescent protein (amino acids 6 to 223 of mScarlet-I3, FPbase ID: THSBS). The sequences of all constructs were confirmed by Sanger sequencing.

### Fusion assay

The fusion assay was performed to determine the effect of restriction factors on the fusogenic activity of Syncytin-1 and Syncytin-2. HEK293T or BeWo cells expressing split-GFP reporter proteins were used (*15*). For HEK293T cells, split-GFP 1-10 were seeded on poly-l-lysine–coated 96-well plates. The next day, the cells were transiently transfected with expression vectors encoding restriction factors (25, 50, or 100 ng, filled up with empty vector DNA) and plasmid encoding Syncytin-1 or Syncytin-2 (1.5 ng) using a standard calcium-phosphate method (*66*). After 6 hours, the culture medium was changed, the HEK293T split-GFP11 cells were seeded on top of the transfected cells in a 1:1 ratio, and the plates were placed in an Incucyte plate reader (Sartorius). Five images per well were taken with ×10 magnification and recorded every 4 hours for up to 48 hours to monitor the cell fusion. The area of GFP-expressing cells was normalized to the total area of cells for each time point.

The BeWo split-GFP fusion assay was performed to analyze the effect of valproate or GBPs on forskolin-triggered cell fusion. To analyze the effect of valproate or IFN-γ on cell fusion, BeWo cells were seeded in a 1:1 ratio and treated with the indicated increasing amounts of valproate (Sigma-Aldrich, catalog no. 676380) or IFN-γ (R&D Systems, catalog no. 285-IF 100). Cell fusion was triggered by addition of 10 μM forskolin (Sigma-Aldrich, catalog no. F6886). To monitor cell fusion, images of the 96-well plate were recorded every 4 hours for up to 72 hours in an Incucyte plate reader (Sartorius). The area of GFP-positive cells was normalized to the total area of cells and integrated over time.

To analyze the effects of *GBP* overexpression on forskolin-triggered cell fusion, BeWo cells were transduced with lentiviral vectors expressing WT or mutated GBPs. To generate the virus stocks for transduction, HEK293T cells were seeded on poly-l-lysine–coated six-well plates and cotransfected with lentiviral-based expression plasmids (for empty vector, GBP2, GBP2-C588A, GBP5, or GBP5-C583A) (1750 ng) together with lentiviral packaging plasmids pMDLg/pRRE (870 ng), pRSV-Rev (440 ng), and pMD2.G (500 ng). In parallel, a lentiviral-based expression plasmid encoding GFP was used to determine transfection and transduction efficiencies rates. The media were changed 16 hours posttransfection, and 48 hours later, the supernatants containing the viral particles were collected and cleared by centrifugation at 1000*g* for 5 min. One day prior transduction, BeWo split-GFP 1-10 and GFP 11 cells were seeded in a 1:1 ratio in 96-well or 6-well plates. The seeded cells were transduced with 50 μl (96-well) or 1 ml (6-well) of virus-containing supernatants. Cell fusion was triggered by adding 20 μM forskolin at the time of transduction. To monitor cell fusion, images of the 96-well plate were recorded every 4 hours for up to 72 hours in an Incucyte plate reader (Sartorius). Three days posttransduction, the cell lysates were collected for Western blot analysis of GBP protein levels (six-well plates).

To analyze the effects of *GBP5* knockout combined with IFN-γ stimulation on forskolin-mediated BeWo cell fusion, the virus stocks for transduction were produced in HEK293T cells in six-well plates. The cells were cotransfected with lentiviral-based lentiCRISPR v2 knockout plasmids (expressing the single guide RNA CATTACGCAACCTGTAGTTG targeting *GBP5* or an empty vector control (Addgene, catalog no. 52961) together with the lentiviral packaging plasmids pMDLg/pRRE (870 ng), pRSV-Rev (440 ng), and pMD2.G (500 ng). The medium was changed 6 hours posttransfection. Twenty-four hours later, lentivirus-containing supernatants were centrifuged at 1000*g* for 5 min and passed through a 0.44-μm filter. These supernatants were then used to transduce BeWo split-GFP cells, which had been seeded 1 day before in T25 flasks. The seeded cells were transduced with the 2.5-ml lentivirus-containing supernatant diluted in a 2.5-ml fresh medium. Two days posttransduction, the BeWo split-GFP cells were seeded in a poly-L-lysine-coated 96-well plate in a 1:1 ratio. In addition, the cells were seeded in 12-well plates for Western blotting. Three days posttransduction, the cells of both plates were stimulated with IFN-γ (100, 10, 1, 0.1, and 0 ng/ml). Five days posttransduction, the cells of the 12-well plate were harvested for Western blotting. The 96-well plate was treated with 20 μM forskolin and then imaged every 4 hours for 44 hours in an Incucyte plate reader.

### Proteolytic processing and glycosylation of Syncytin-1 and Syncytin-2

To analyze the cleavage of Syncytins, HEK293T cells were cotransfected with expression plasmids for V5-tagged Syncytin-1 (2 μg) or Syncytin-2 (1 μg) and increasing amounts of expression plasmids for HA-tagged WT or isoprenylation-deficient GBPs (0, 0.75, 1.5, and 2.5 μg). Two days posttransfection, Syncytin cleavage in the presence or absence of GBPs was analyzed by Western blotting.

To analyze alterations in the N-linked glycosylation pattern of Syncytin-1, HEK293T cells were cotransfected with an expression plasmid for V5-tagged Syncytin-1 (2 μg) and expression plasmids for GBP2 or GBP5 (2.5 μg). Cell lysates were either left untreated or treated with PNGase F (NEB) according to the manufacturer’s protocol. The N-linked glycosylation pattern of Syncytin-1 was analyzed by Western blot.

### Quantitative reverse transcription PCR

To monitor *GBP5* expression, BeWo cells were stimulated with IFN-γ or the respective solvent control. Twenty-four hours or 48 hours poststimulation, cells were harvested, and total RNA was isolated using the RNeasy Mini Kit (QIAGEN, catalog no. 74106) following the manufacturer’s instructions. RNA quality and quantity were assessed using a spectrophotometer. Reverse transcription was performed using the PrimeScript RT Reagent Kit (Perfect Real Time) (TAKARA, catalog no. RR037A) with oligo dT primers and random hexamers. Quantitative real-time PCR was conducted using specific primer/probe sets for *GBP5* (Thermo Fisher Scientific, catalog no. Hs00369472_m1), with glyceraldehyde phosphate dehydrogenase (GAPDH; Thermo Fisher, catalog no. Hs02786624_g1) serving as the internal control. Each sample was analyzed in technical triplicates.

### Western blot

To determine protein levels, cells were washed with phosphate-buffered saline (PBS), lysed in Western blot lysis buffer [150 mM NaCl, 50 mM Hepes, 5 mM EDTA, 0.1% NP-40, 500 mM Na_3_VO_4_, and 500 mM NaF (pH 7.5)] containing protease inhibitors for 45 min on ice. The cell lysates were then cleared by centrifugation at 10,000*g* for 5 min at 4°C. Lysates were mixed with protein sample loading buffer (LI-COR, catalog no. 928-40004) supplemented with 10% β-mercaptoethanol and heated at 95°C for 5 min. Proteins were separated on NuPAGE 4 to 12% bis-tris Gels (Thermo Fischer Scientific, catalog no. NP0323BOX), blotted onto Immobilon-FL polyvinylidene difluoride membranes (Merck Millipore, catalog no. IPFL00010), and blocked by 5% milk in PBS containing 0.2% Tween 20. All washing and steps were performed using PBS containing 0.2% Tween 20. The membranes were stained using primary antibodies directed against HA tag (Abcam, catalog no. ab18181), AU1-tag (Novus Biologicals, catalog no. NB600-453), V5-tag (Cell Signaling, catalog no. 13202), GBP5 (Santa Cruz, catalog no. sc-160353), furin (R&D Systems, catalog no. AF1503), GAPDH (BioLegend, catalog no. 607902), and infrared dye-labeled secondary antibodies (LI-COR IRDye). Proteins were detected using a LI-COR Odyssey scanner, and band intensities were quantified using LI-COR Image Studio Lite version 5.2.

### AMC reporter assay

To determine PCSK activity in HEK293T cells, 700,000 cells per well were seeded on six-well plates 1 day before transfection. HEK293T cells were transfected with expression plasmids encoding furin, PCSK5, PCSK6, or PCSK7 (5 or 2.5 μg), either alone or in combination with GBPs (2.5 μg) using a standard calcium phosphate transfection method. One day posttransfection, the cells were harvested, washed with PBS, and lysed with AMC lysis buffer [500 mM Hepes, 5 mM CaCl_2_, 5 mM β-mercaptoethanol, and 2.5% Triton X-100 (pH 7)] for 20 min on ice. The lysates were cleared by centrifugation at 20,000*g* for 15 min at 4°C.

An AU1 antibody (Novus Biologicals, catalog no. NB600-453) and its isotype control (Abcam, catalog no. ab172730) were diluted in antibody dilution buffer [50 mM Na_2_CO_3_ (pH 9.6)] to a final concentration of 10 ng/μl. Fifty microliters of the diluted antibodies was aliquoted per well of an enzyme-linked immunosorbent assay (ELISA) plate (Sarstedt 82.1581.200). The plate was sealed and incubated at room temperature overnight in a dark chamber. The following day, the antibody coating solution was discarded, and the plate was washed three times with washing buffer (PBS containing 0.05% Tween 20). The wells were blocked by addition of blocking solution (PBS containing 0.5% sucrose and 0.5% Tween 20) and incubated for 4 hours in the dark at room temperature. After blocking, the wells were washed thrice using the washing buffer. Last, the cell lysates were added to the wells, and the plate was incubated overnight at 4°C on a shaker. The next day, the lysates were discarded, and the wells were washed thrice with the washing buffer. Twenty microliters of 1 mM CaCl_2_ was added to the wells and incubated with 80 μl of pyruvyl (Pyr)– or succinyl (Suc)–modified 1 mM AMC substrates (control, Pyr-RTKR-AMC; Syn-1, Suc-VISKPRNKR-AMC; Syn-2, Suc-NSPLPRVRR-AMC; Syn-2-P2, Suc-NSPLPRVKR-AMC; Syn-2-P3, Suc-NSPLPRNRR-AMC; Syn-2-P6, Suc-NSPKPRVRR-AMC; Syn-2-P2/3, Suc-NSPLPRNKR-AMC; Syn-P2/6, Suc-NSPKPRVKR-AMC; Syn-2-P3/6, Suc-NSPKPRNRR-AMC; Syn-2-P2/3/6, Suc-NSPKPRNKR-AMC [all from Leon (Nanjing) Biotechnology]. Substrate cleavage was monitored for 5 hours with measurements recorded in 2-min intervals using a Cytation3 imaging reader (BioTek; 355-nm excitation and 460-nm emission) at 37°C. Postmeasurement, the supernatants were discarded, 40 μl of 1× protein sample loading buffer (LI-COR) was added to the wells, and the plate was sealed and incubated on a heat shaker at 95°C for 10 min. Samples were transferred to microfuge tubes and heated again 95°C for 5 min before loading on an SDS gel for Western blot analysis.

For the AMC assay with recombinant PCSK7/furin, 50 ng of the respective recombinant protease [furin (R&D Systems, catalog no. 1503-SE-010) or PCSK7 (R&D Systems, catalog no. 2984-SE-010)] in 1 mM CaCl_2_ was aliquoted per well of an ELISA plate (Sarstedt 82.1581.200). AMC substrate (1 mM; described above) was added to the wells, and substrate cleavage was monitored at 37°C for 5 hours using a Cytation3 imaging reader.

### Coimmunoprecipitation

To investigate possible interactions between GBPs and furin and/or PCSK7, coimmunoprecipitation with subsequent Western blot analysis was performed. Briefly, HEK293T cells were seeded in six-well plates and cotransfected with expression plasmids for HA-tagged GBPs and AU1-tagged furin or PCSK7 (ratio 1:1; total of 5 μg DNA per well). One day posttransfection, cells were lysed in Western blot lysis buffer and cleared by centrifugation (see the “Western blot” section). Forty microliters of the lysate was used for whole-cell lysate analysis and further prepared as described in the “Western blot” section, while the rest of the lysate was used for coimmunoprecipitation. A preclearing step was performed to remove unspecifically bound proteins from the lysate. Pierce Protein A/G magnetic beads (Thermo Fisher Scientific, catalog no. 88802) were washed three times with 1 ml of NP-40 wash buffer [50 mM Hepes, 300 mM NaCl, and 0.5% NP-40 (pH 7.4)] and added to the lysate. After incubation for 1 hour at 4°C, beads were removed from the lysate using a magnetic rack. To precipitate protein complexes, the lysate was incubated first with an anti–HA- or anti–AU1-tag antibody (2 μg per sample) overnight at 4°C. The next day, 10 μl of washed Protein A/G magnetic beads was added to the antibody-lysate mix and incubated at 4°C for 4 hours. After incubation, the beads were washed three times in NP-40 wash buffer before incubation with 100 μl of 1× protein sample loading buffer (LI-COR) at 95°C for 10 min to recover bound proteins. The whole-cell lysates and precipitates were analyzed by Western blot.

### Infectivity of virions pseudotyped with Syncytin

To analyze the effects of GBP2 and GBP5 on Syncytin-mediated infectious virus yield, VSVΔG-GFP or avian alpharetroviral particles were pseudotyped with Syncytin-1 or Syncytin-2. For VSVΔG-GFP analysis, HEK293T cells were cotransfected with expression plasmids for Syncytin-1 or Syncytin-2 (0.5 μg) and increasing amounts of GBP2, GBP5, GBP2 C588A, or GBP5 C583A (0, 0.75, 1.5, and 2.5 μg). Twenty-four hours posttransfection, cells were infected with VSVΔG-GFP particles pseudotyped with VSV-G at a multiplicity of infection of 3. The inoculum was removed 1 hour postinfection. Syncytin-pseudotyped VSVΔG-GFP particles were harvested 16 hours postinfection, and cell debris was removed by centrifugation for 3 min at 950*g*. Subsequently, 10% (v/v) of the I1-hybridoma supernatant was added to the cell culture supernatant to block the remaining input particles carrying VSV-G. One day before this, HEK293T cells were seeded on a 96-well plate (21,000 cells per well). These cells were infected in triplicates with 100 μl of l1-hybridoma blocked supernatants containing Syncytin VSVΔG-GFP pseudovirions. The cells were fixed 18 hours postinfection in 2% paraformaldehyde for 30 min at 4°C. Last, GFP-positive cells, i.e., infected cells, were quantified using a CANTO II flow cytometer.

The alpharetroviruses pseudotyped with Syncytin-1 or Syncytin-2 envelope glycoproteins were prepared by cotransfection of MFSD2A knockout HEK293T cells, which, because of the lack of MFSD2A receptor, showed higher Syncytin-2 pseudotype production than WT HEK293T cells. One day before transfection, 600,000 cells were seeded on P35 dishes. On the next day, the cells were transfected with Lipofectamine 3000 (Thermo Fisher Scientific) according to the manufacturer’s instructions. The first half (1.25 μg) of the transfection mix contained plasmids encoding alpharetroviral gagpol, pAlpha-SF-mScarlet-wPRE, and plasmids encoding envelope glycoproteins in the ratio 25:49:1. The second half (1.25 μg) contained plasmids encoding GBP2, GBP5, GBP2 C588A, GBP5 C583A, or IFITM3 (0, 12.5, 125 ng, or 1.25 μg, filled up with empty vector DNA). Four hours after transfection, the medium was changed for 2.5 ml of fresh media. Two days posttransfection, the supernatant containing virus particles was collected and filtered through 0.45-μm filters, and the cells were pelleted for Western blot analysis.

To improve the detection of Syncytin-2–pseudotyped viruses, HEK293T cells were modified by transduction to ectopically express human MFSD2A fused with GFP. The transducing virus was prepared by cotransfection of HEK293T cells as previously described (*33*).

To determine restriction of virus production, 50,000 cells were seeded in 48-well plates. On the next day, the cells were infected with 200 μl of virus supernatants and spinoculated at 1200*g* for 1.5 hours at 25°C. After spinoculation, the medium was exchanged. On the third day after infection, the cells were detached with trypsin-EDTA solution, fixed in paraformaldehyde (1% final concentration), and analyzed by a FACSymphony (BD) flow cytometer. Infected cells were positive for mScarlet (excitation laser: 561 nm, emission filter: 610/20 nm).

### Single-cell RNA sequencing data

To analyze the expression of PCSKs in primary trophoblast cells, we took advantage of a previously published RNA sequencing dataset by Vento-Tormo and colleagues (*12*), who analyzed first trimester placental tissue. Cell types were annotated, and expression levels were visualized using the Single Cell Expression Atlas and the Human Protein Atlas (*32*).

### Disease association analyses

Differential gene expression in placenta samples from patients suffering from PE or FGR versus healthy placenta samples were derived from publicly available datasets generated and published by Nishizawa *et al*. (*24*) (Gene Expression Omnibus accession number GSE24129) (Fig. 1, C and D) and Zhou *et al*. (*34*) (fig. S2) (GSE40182) via the Gene Expression Browser. For Fig. 1 (C and D), genes with the highest *n* fold change were selected, irrespective of statistical significance. No human subjects were recruited, and no new patient material was acquired for the present study.

### Statistical analysis

All statistical calculations were performed using GraphPad PRISM 10.1.1 (GraphPad Software). *P* values were determined using one-way analysis of variance (ANOVA) with Dunnett’s multiple comparisons test or paired *t* test. Unless otherwise stated, data are shown as the mean of at least three independent experiments ± SD with each data point representing one biological replicate. Significant differences are indicated as *P < 0.05, **P < 0.01, ***P < 0.001, and ****P < 0.0001.
