# Supplementary material for: Inhibition of placental trophoblast fusion by guanylate-binding protein 5
Source: Sci Adv. 2025 May 7;11(19):eadt5388. doi: 10.1126/sciadv.adt5388 (PMC12057675; doi:10.1126/sciadv.adt5388)
Supplement: Supplementary file 1 — Figs. S1 to S8 [file sciadv.adt5388_sm.pdf]

Science Advances
AAAS

# Supplementary Materials for

## Inhibition of placental trophoblast fusion by guanylate-binding protein 5

Veronika Krchlikova *et al.*

Corresponding author: Daniel Sauter, daniel.sauter@med.uni-tuebingen.de

*Sci. Adv.* **11**, eadt5388 (2025)
DOI: 10.1126/sciadv.adt5388

**This PDF file includes:**

Figs. S1 to S8

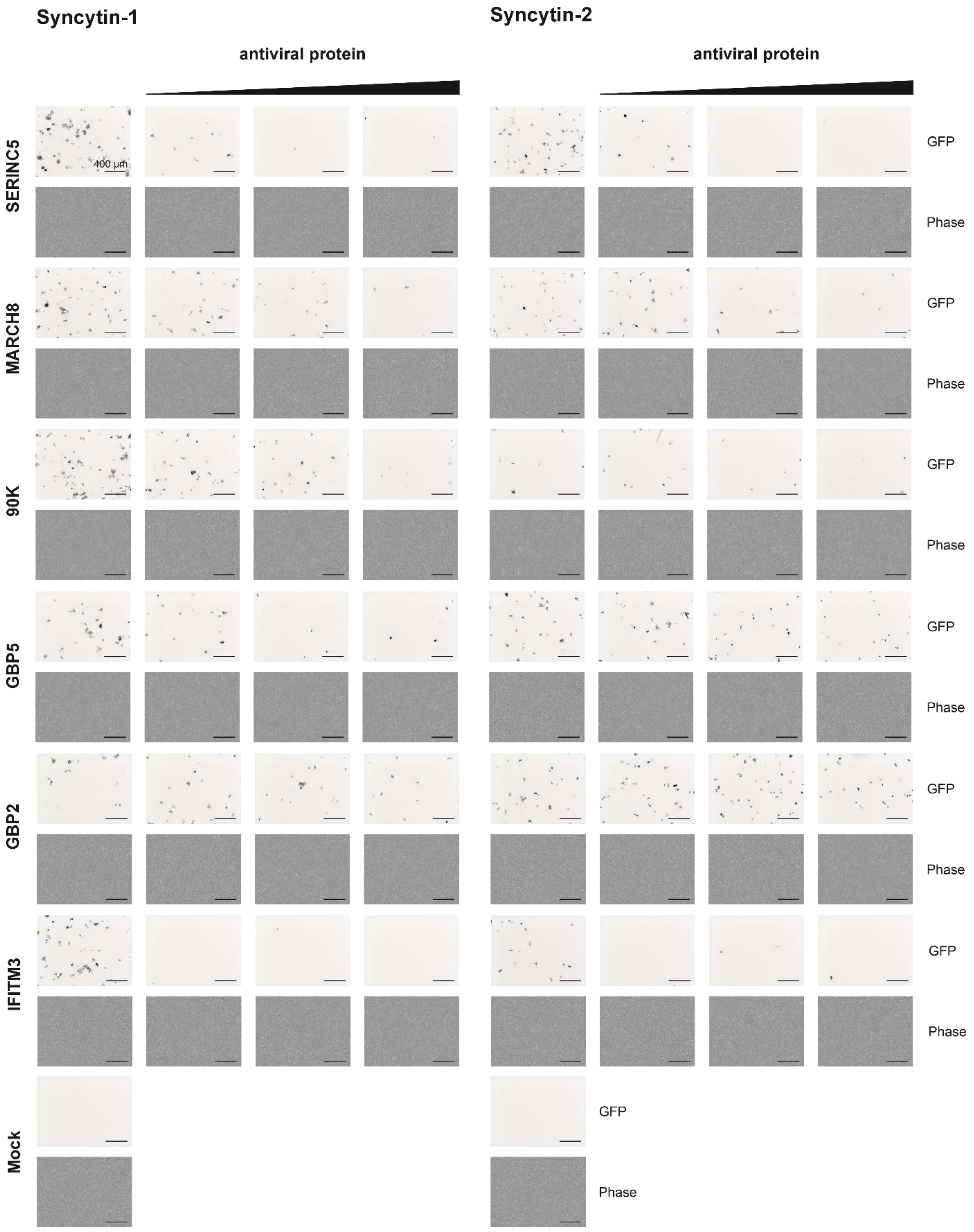
Syncytin-1
Syncytin-2
antiviral protein
antiviral protein
SERINC5
MARCH8
90K
GBP5
GBP2
IFITM3
Mock
400 µm
GFP
Phase

**Fig. S1.**

**Antiviral proteins inhibit Syncytin-mediated cell fusion.** HEK239T cells stably expressing the N-terminal portion of GFP were co-transfected with expression plasmids for Syncytin-1 or Syncytin-2 and increasing amounts of plasmids expressing the indicated antiviral proteins (i.e. SERINC5, MARCH8, 90K, GBP5, GBP2, and IFITM3) and mixed with HEK293T cells expressing the C-terminal portion of GFP. GFP fluorescence was quantified every 4 hours over a period of 48 hours as a marker for syncytia formation. Exemplary images of data analyzed in Fig. 1B are shown. GFP represents syncytia formation and phase contrast (Phase) displays cell density. Images obtained 48 h post transfection are shown (scale bar = 400 µm).

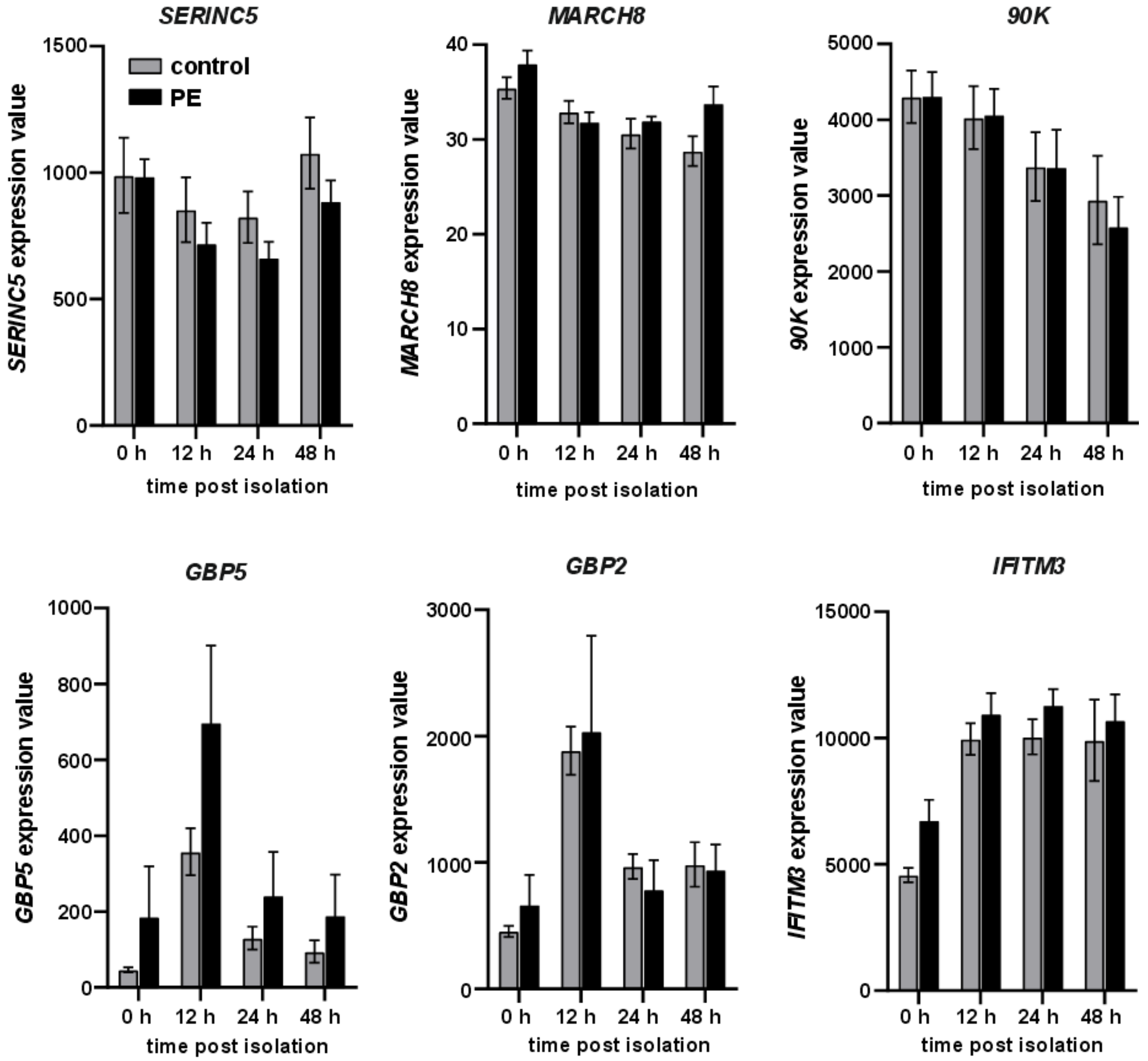


**Fig. S2.**

**Antiviral gene expression in *ex vivo* cultured cytotrophoblast cells isolated from preeclamptic pregnancies**. Data are derived from GSE40182 via the Gene Expression Browser (GXB) and have been described by Zhou and colleagues (34). Briefly, cytotrophoblast cells were isolated from preterm placentas of women suffering from severe preeclampsia (n=5, mean ± SEM) and cultured *ex vivo*. Placentas of preterm labor patients without preeclampsia and without any signs of infection (n=4-5, mean ± SEM) served as gestation-matched controls. At the indicated time points post isolation, cells were harvested, and microarray analyses were performed. Relative expression values of the antiviral factors *SERINC5*, *MARCH8*, *90K*, *GBP5*, *GBP2* and *IFITM3* in control (grey) vs. pre-eclamptic (PE, black) samples are shown.

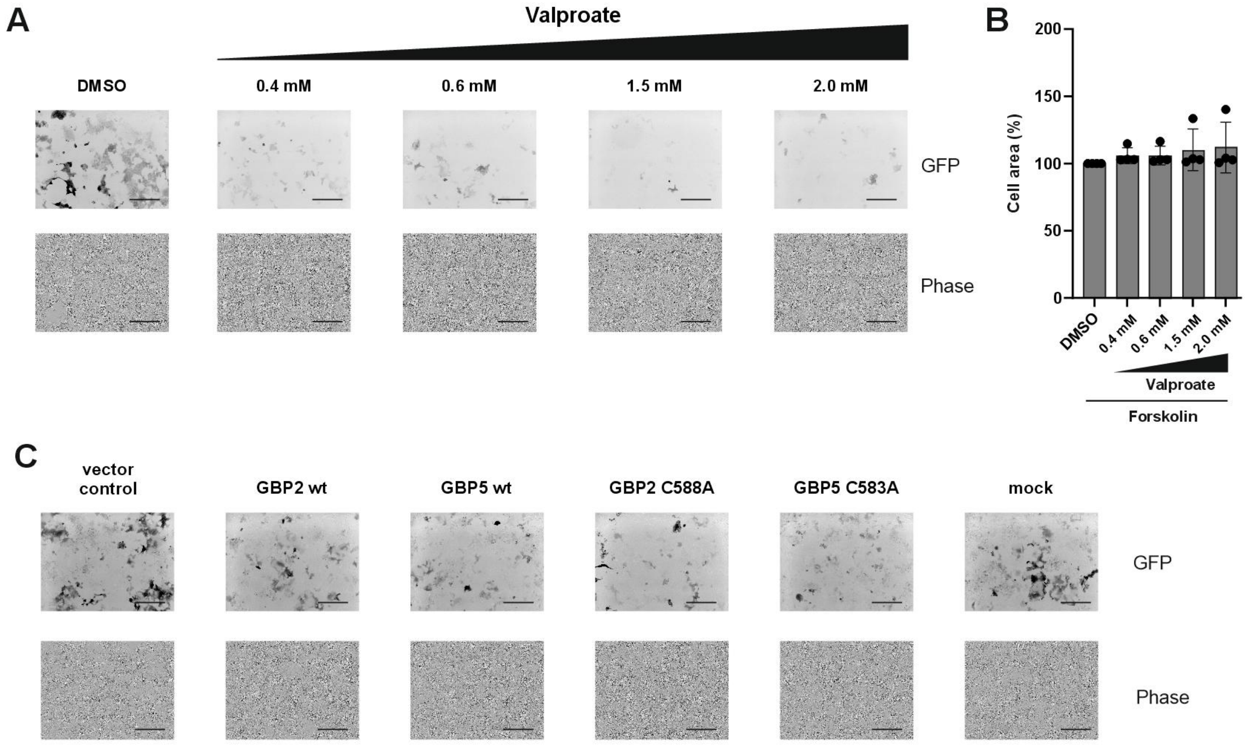


**Fig. S3.**

**Antiviral proteins and valproate inhibit BeWo cell fusion.** (**A**) BeWo cell fusion upon valproate stimulation. Exemplary images of data analyzed in Fig. 1G are shown. GFP represents syncytia formation, and phase contrast (Phase) displays cell density. Images obtained 72 h post transfection are shown (scale bar = 400 µm). (**B**) Total area of valproate-treated BeWo cells analyzed in Fig. 1G was determined and normalized to the DMSO control. Mean values of 4 independent experiments ± SD are shown. (**C**) BeWo cell fusion after exogenous GBP2/5 expression. BeWo split-GFP cells were mixed and transduced with wild type or mutated GBPs. Immediately after transduction, cell fusion was induced by forskolin stimulation, and GFP fluorescence was monitored over a period of 72 hours. Exemplary images of data analyzed in Fig. 1J are shown. GFP represents syncytia formation and phase contrast (Phase) displays cell density. Images obtained 72 h post transfection are shown (scale bar = 400 µm).

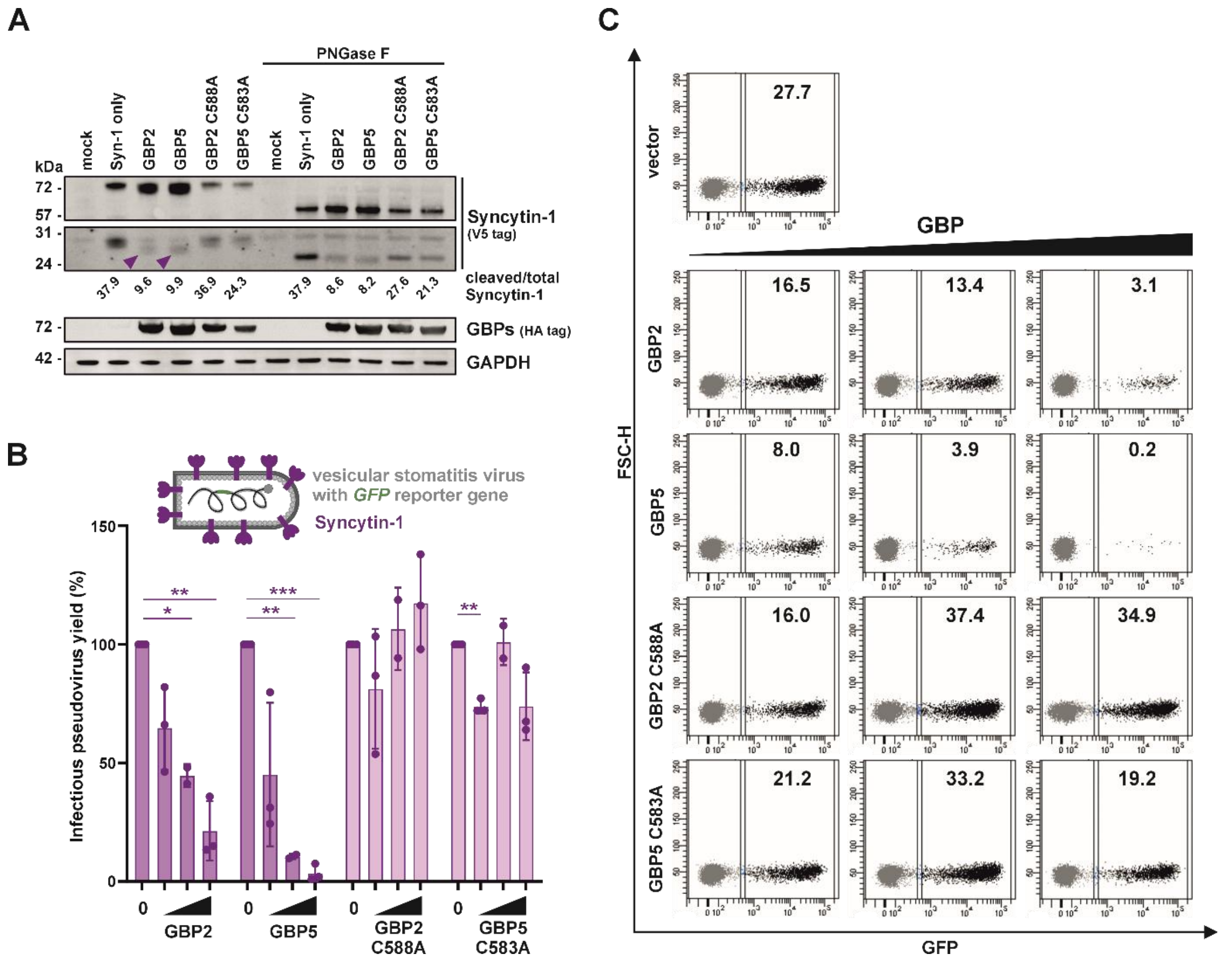


**Fig. S4.**

**GBP2 and GBP5 interfere with the N-linked glycosylation and fusogenic activity of Syncytin-1.** (**A**) Syncytin-1 glycosylation in the presence of GBPs. HEK293T cells were co-transfected with expression plasmids for V5-tagged Syncytin-1 and HA-tagged GBP2 or GBP5. Two days post-transfection, cells were harvested for Western blotting. One half of the samples was treated with PNGase F before blotting to remove N-linked oligosaccharides. One representative Western blot is shown. Purple arrow heads indicate the shift in the electrophoretic mobility of Syncytin. Cleavage of Syncytin-1 was determined by calculating the amount of cleaved to total Syncytin-1. (**B, C**) Effect of GBPs on the infectivity of Syncytin-1-pseudotyped VSV. GFP-expressing VSVΔG pseudotyped with Syncytin-1 was generated in HEK293T cells expressing increasing amounts of GBPs or the respective negative control (vector). Infectious pseudovirus yield was quantified by infecting HEK293T cells and determining the percentage of GFP positive cells. Black triangles indicate increasing amount of transfected antiviral protein. Mean values of two to four experiments ± SD are shown in (B), exemplary FACS plots are shown in (C). A one-way ANOVA with multiple comparisons (Dunnett´s test) was performed (* $p<0.05$; ** $p<0.01$; *** $p<0.001$).

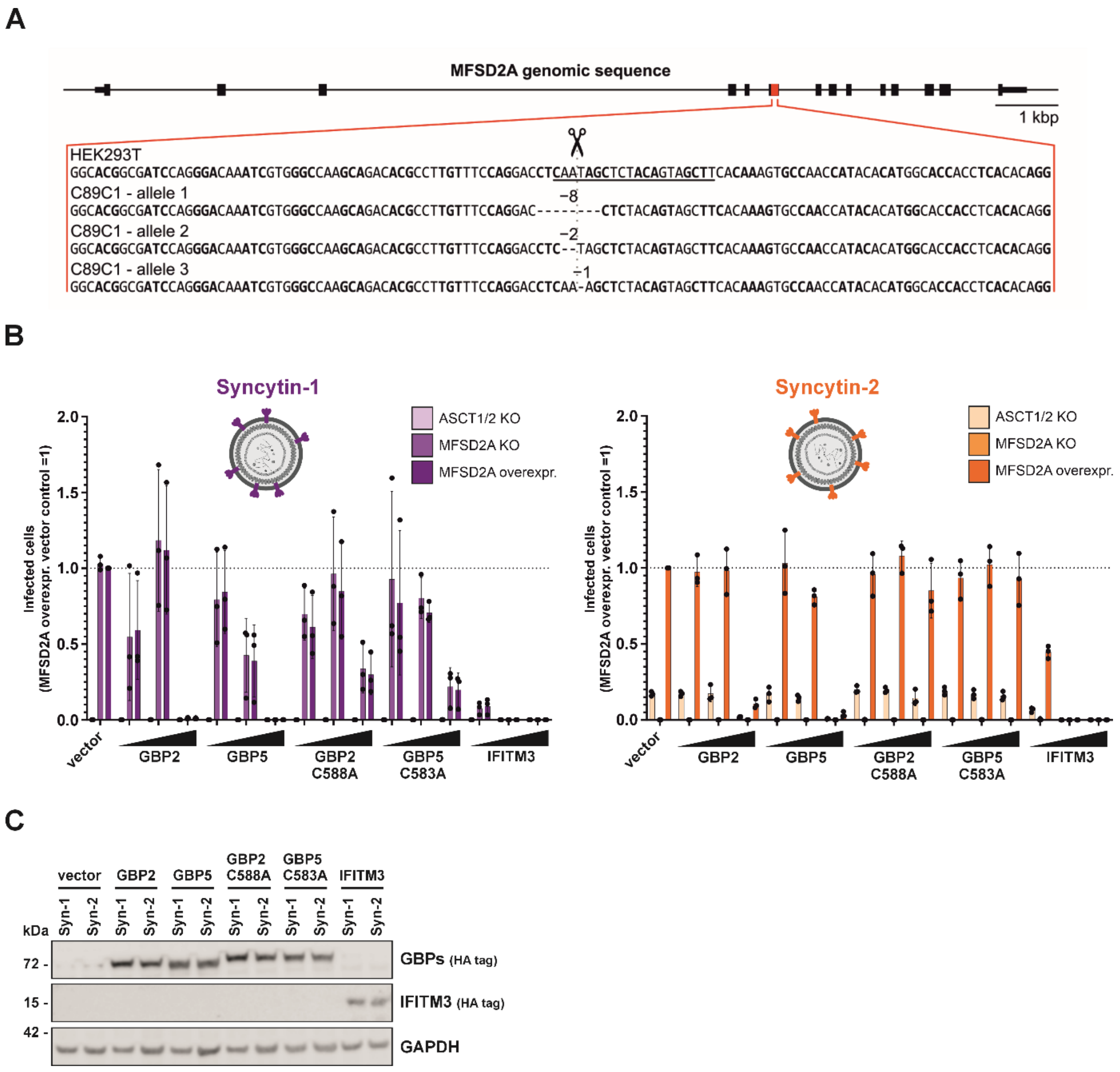


**Fig. S5.**

**Generation of *MFSD2A* knockout HEK293T cells and effect of GBP2/5 on the fusogenic activity of Syncytins.** (**A**) Characterization of the *MFSD2A* knockout in HEK293T cells. The CRISPR-Cas9 target sequence (gRNA) in *MFSD2A* exon 6 is underlined. The three alleles in HEK293T cells with their respective genotypes are depicted below with 8, 2 and 1 nucleotide deletions, respectively. (**B**) Infectivity of Syncytin-carrying pseudovirions in the presence of GBPs. *MFSD2A* knock-out HEK293T cells were co-transfected with expression plasmids for Syncytin-1 or -2 together with ASLV gag-pol, mScarlet minigenome and GBP or IFITM3. Two days later, supernatants were collected and used for the infection of naïve HEK293T cells overexpressing MFSD2A, ASCT1/2 knockout cells and *MFSD2A* knockout cells. Three days post infection, the percentage of infected cells was determined by flow cytometry. Black triangles indicate increasing amount of transfected antiviral protein. Mean values of three independent experiments ± SD are shown. (**C**) Western blot analysis of transfected HEK293T cells producing Syncytin-pseudotyped virions (connected to Fig. 2E). The cells for Western blotting were collected at the same time as the supernatants. One representative Western blot of HEK293T cells transfected with middle amount of expression plasmid for the respective antiviral protein is shown.

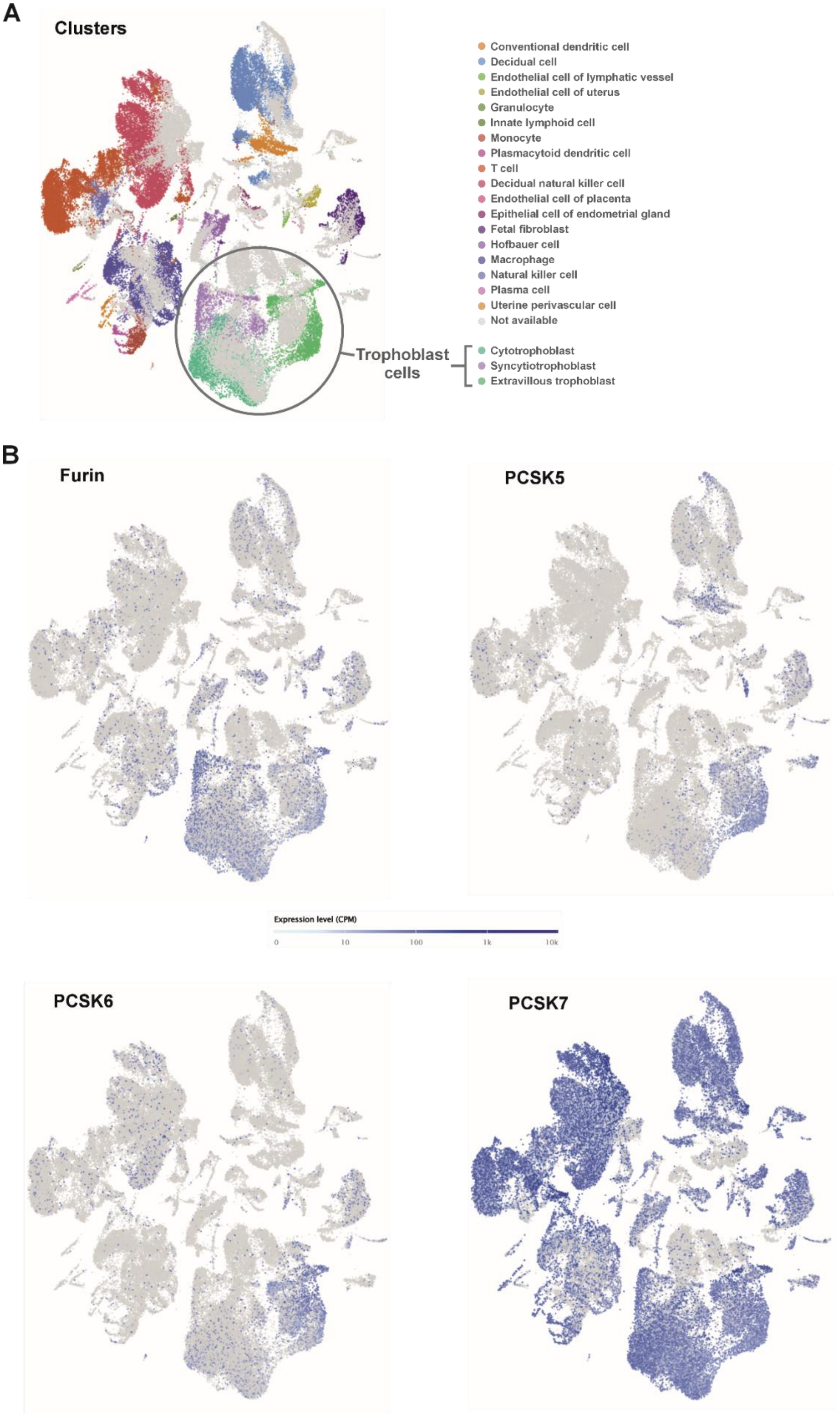
A
Clusters
Conventional dendritic cell
Decidual cell
Endothelial cell of lymphatic vessel
Endothelial cell of uterus
Granulocyte
Innate lymphoid cell
Monocyte
Plasmacytoid dendritic cell
T cell
Decidual natural killer cell
Endothelial cell of placenta
Epithelial cell of endometrial gland
Fetal fibroblast
Hofbauer cell
Macrophage
Natural killer cell
Plasma cell
Uterine perivascular cell
Not available
Trophoblast cells
Cytotrophoblast
Syncytiotrophoblast
Extravillous trophoblast
B
Furin
PCSK5
Expression level (CPM)
0
10
100
1k
10k
PCSK6
PCSK7

**Fig. S6.**

**Expression of furin, PCSK5, 6 and 7 in placental cells.** Single cell expression levels of furin and PCSK5-7 from healthy first trimester placental tissue were obtained from Vento-Tormo, *et al.* (12) via the Human Protein Atlas (**A**) Uniform manifold approximation and projection (UMAP) clustering of cell types; cell types were annotated in the Single Cell Expression Atlas. (**B**) Expression of furin, PCSK5, 6 and 7 in single cells, is presented as counts per million (CPM) and overlaid on the UMAP map.

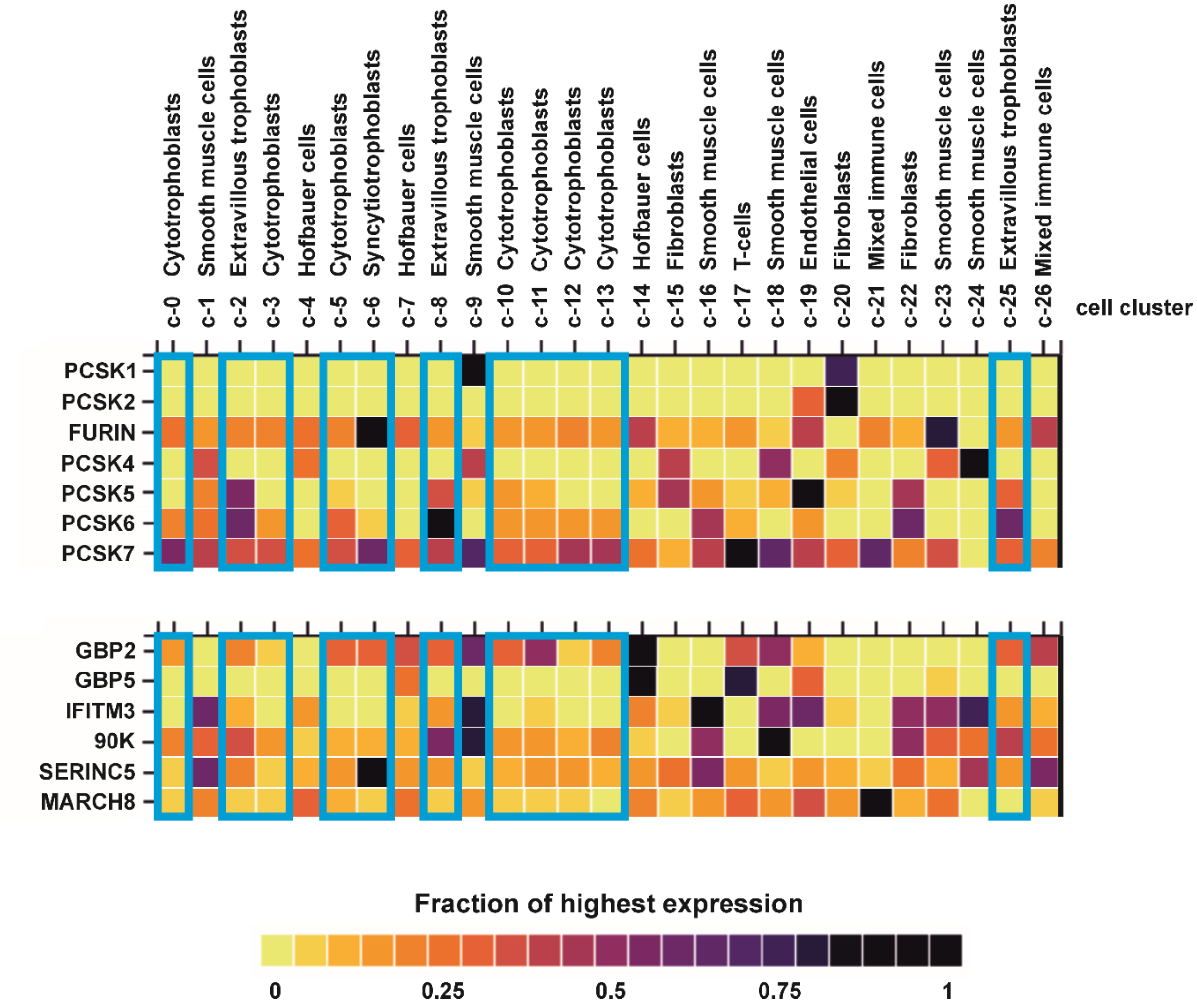


**Fig. S7.**

**Expression of PCSK1-7 and antiviral proteins in placental cells.** Single cell expression levels of PCSK1-7 and antiviral proteins in healthy first trimester placental tissue were obtained from Vento-Tormo, *et al.* (12) via the Human Protein Atlas. The heat map shows RNA expression in each cell type cluster as normalized transcripts per million. Some cell types formed separate subclusters defined by differential expression of specific marker genes (see Human Protein Atlas for details). In the upper part, expression of PCSK1-7 is shown. In the lower part, expression of antiviral proteins is shown. Trophoblast cell clusters are highlighted in blue.

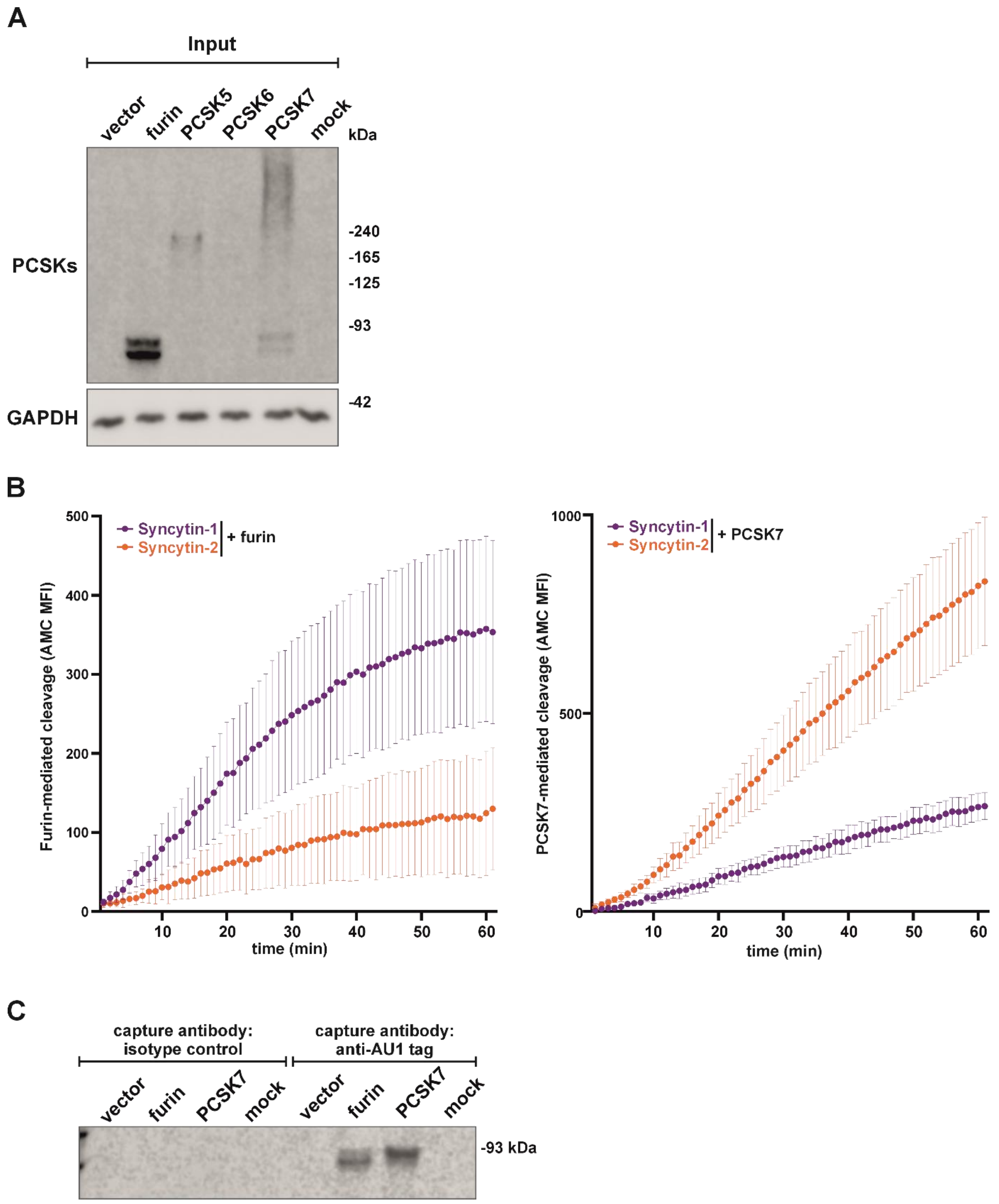


**Fig. S8.**

**Cleavage of Syncytins by furin and PCSK7.** (**A**) Analysis of exogenous PCSK expression in HEK293T cells. The cells were transfected as described in Fig. 3B. The cell lysates were analyzed by Western blotting. A representative Western blot from data analyzed in Fig. 3B is shown. (**B**) Syncytin cleavage by recombinant PCSKs produced in myeloid cells. The indicated AMC reporter substrates were incubated with furin (left panel) or PCSK7 (right panel). Substrate cleavage was monitored over a period of 60 min. Mean values of 3 technical replicates ± SEM are shown. (**C**) Capture efficiency of PCSKs in the experiment described in Fig. 3F was monitored by Western blotting. One exemplary Western blot is shown.
